# Respiratory Outcomes After Transcatheter vs Surgical Patent Ductus Arteriosus Closure in Preterm Infants

**DOI:** 10.1001/jamanetworkopen.2025.13366

**Published:** 2025-06-03

**Authors:** Valerie Y. Chock, Shazia Bhombal, Alexis S. Davis, Meera N. Sankar, Barbara T. Do, Matthew M. Laughon, Krisa P. Van Meurs, Carl H. Backes, Patrick J. McNamara

**Affiliations:** 1 Division of Neonatal and Developmental Medicine, Stanford University School of Medicine, Palo Alto, California; 2 Department of Pediatrics, Emory University School of Medicine, Atlanta, Georgia; 3 Social, Statistical and Environmental Sciences Unit, RTI International, Rockville, Maryland; 4 Department of Pediatrics, University of North Carolina at Chapel Hill, Chapel Hill; 5 The Heart Center, Nationwide Children’s Hospital, Columbus, Ohio; 6 Department of Pediatrics, University of Iowa, Iowa City

## Abstract

**Importance:**

Transcatheter closure of the patent ductus arteriosus (PDA) has increasingly been adopted in extremely preterm infants as a method to definitively close the PDA while avoiding the inherent risks of surgical ligation. Differences in respiratory outcomes after transcatheter closure compared with surgical ligation have not been substantiated, particularly in the context of timing of the intervention.

**Objective:**

To characterize respiratory outcomes in extremely preterm infants with PDA treated with transcatheter device closure compared with surgical ligation.

**Design, Setting, and Participants:**

This retrospective cohort study assessed data from preterm infants born at less than 29 weeks’ gestation or with birth weight less than 1000 g who underwent definitive PDA closure in neonatal intensive care units participating in the Neonatal Research Network’s Generic Database between January 1, 2016, and December 31, 2020. Data were analyzed from October 2021 to February 2024.

**Exposures:**

PDA treatment with transcatheter device closure or with surgical ligation.

**Main Outcomes and Measures:**

The primary outcome was total days of mechanical ventilation.

**Results:**

Of 3806 included infants with a PDA diagnosis, 202 underwent transcatheter PDA closure (median [IQR] gestational age, 25.4 [24.1-27.1] weeks; 114 [56%] female) and 359 underwent surgical ligation (median [IQR] gestational age, 24.9 [24.0-25.9] weeks; 187 [52%] female). Infant age at transcatheter closure was older than at surgical ligation (mean [SD], 58.7 [28.4] vs 33.6 [16.7] days; *P* < .001). After adjustment of analyses for center, birth year, gestational age, age at PDA intervention, and prior pharmacologic treatment, infants with transcatheter closure compared with surgical ligation had comparable respiratory outcomes, including total days of mechanical ventilation (adjusted median difference, −2.65 [95% CI, −8.36 to 3.07] days; *P* = .36).

**Conclusions and Relevance:**

In this cohort study of extremely preterm infants who underwent transcatheter closure compared with surgical ligation for treatment of PDA, respiratory outcomes did not differ, although the transcatheter closure group had a longer duration of PDA exposure. Future research evaluating outcomes after transcatheter PDA closure should assess the optimal timing of definitive intervention.

## Key Points

**Question:**

Do respiratory outcomes differ after transcatheter closure of the patent ductus arteriosus (PDA) compared with surgical ligation in extremely preterm infants?

**Findings:**

In this cohort study of 202 extremely preterm infants who underwent transcatheter closure and 359 who underwent surgical ligation, respiratory outcomes, including total days of mechanical ventilation, did not differ by PDA treatment, despite a longer duration of PDA exposure in the transcatheter closure group.

**Meaning:**

These findings suggest that similar respiratory outcomes may be anticipated after transcatheter PDA closure compared with surgical ligation, although future research evaluating outcomes will require optimization of timing of definitive PDA intervention.

## Teaser

This cohort study compares respiratory outcomes after treatment of patent ductus arteriosus with transcatheter closure vs surgical ligation among extremely preterm infants.

## Introduction

Transcatheter (percutaneous) closure of the patent ductus arteriosus (PDA) has been increasingly used across centers with advances in technology and optimized venous retrograde approaches. The frequency of life-threatening events appears to be relatively rare, even in preterm infants weighing less than 1.5 kg.^1,2,3^ Risks, including death, device migration, device malposition, inability to place the device, cardiovascular injury, and complications related to anesthesia, have been reported.^4,5,6^ The US Food and Drug Administration approval of a percutaneous device for use in premature infants weighing more than 700 g as early as postnatal day 3 occurred in 2019.^3^ The indications and optimal timing for transcatheter PDA closure, however, remain unclear. Further characterization of the population undergoing transcatheter closure is a research priority; specifically, investigating factors such as the timing of intervention and prior pharmacologic treatment is essential to identify infants most likely to benefit.

Comparison with other PDA management strategies, including surgical ligation, medical management, and conservative treatment, is essential to informing the optimal approach to the PDA. Secular changes in the approach to PDA include a reduction in the rate of surgical ligation but an increase in transcatheter closure rates.^7,8,9,10,11^ Inherent risks associated with surgery, including postligation cardiac syndrome (PLCS), impaired cerebral autoregulation, and potential risks of neurosensory impairment,^12,13,14^ may factor into decisions to avoid surgical ligation. Although infants treated with both techniques are likely similar regarding the hemodynamic relevance of their PDA, direct comparisons of respiratory outcomes between groups are limited, and no study, to our knowledge, of preterm infants has focused on total days of mechanical ventilation as a primary outcome or whether the timing of PDA intervention is associated with respiratory outcomes.

With the ongoing controversies and changing landscape of PDA management, investigation of large neonatal databases with transcatheter PDA closure information is critical to explore patient outcomes and help inform the design of future PDA clinical trials.^15^ The present study explored the Generic Database (GDB) of the Eunice Kennedy Shriver National Institute of Child Health and Human Development Neonatal Research Network (NRN) to test the hypothesis that preterm infants undergoing transcatheter PDA closure would have fewer total ventilator days and improved secondary outcomes, including reduction in days of positive pressure ventilation, shorter length of stay, and less need for home oxygen, compared with preterm infants with PDA treated using surgical ligation.

## Methods

### Study Population

This retrospective cohort study used prospectively collected data from the NRN GDB, a registry of extremely preterm infants with birth weights of 401 g to 1000 g and/or gestational age of 22 weeks 0 days through 28 weeks 6 days. This study using the GDB was approved by the institutional review board at each of the 18 participating sites. Per individual institutional review board requirements, data were collected under a waiver of consent or after informed consent was obtained from parents or legal guardians. Prespecified variables from medical records were entered by trained study coordinators. Data from January 1, 2016, until December 31, 2020, were used to compare infants with transcatheter PDA closure with infants with PDA treated using surgical ligation. Specific data on transcatheter PDA closure have been collected in this database since 2016. The Strengthening the Reporting of Observational Studies in Epidemiology (STROBE) reporting guideline for observational studies was followed for this study.

Infants were included in the study if they had a PDA diagnosis and were treated with either transcatheter closure or surgical ligation during their initial hospital admission after birth. Infants were excluded if they were outborn or had congenital malformations or syndromes. PDA diagnosis was defined as clinical evidence of left-to-right PDA shunt documented by any of the following: continuous murmur, hyperdynamic precordium, bounding pulses, wide pulse pressure, congestive heart failure, increased pulmonary vasculature or cardiomegaly observed by chest radiography, increased oxygen requirement, or echocardiography evidence of PDA with left-to-right ductal shunting. PDA treatment characteristics were assessed, including age at intervention. Previous exposure to pharmacologic PDA therapies, specifically with indomethacin, ibuprofen, or acetaminophen, was identified given the potential associations between medications and shunt size or respiratory outcomes.

### Clinical Data Collected

Maternal data, including race and ethnicity,^16^ chorioamnionitis, preeclampsia, antenatal steroid administration, and mode of delivery were collected. Participant race and ethnicity were based on self-report from the electronic health record and were assessed in this study to more fully characterize the cohort. Race categories included Black, White, and other, which comprised American Indian or Alaska Native, Asian, Native Hawaiian or Other Pacific Islander, or more than 1 race. Ethnicity categories included Hispanic and non-Hispanic. Neonatal data were collected from birth until death, discharge home, transfer, or 120 days and included the morbidities of spontaneous gastrointestinal tract perforation; culture-positive sepsis; necrotizing enterocolitis (NEC), defined as modified Bell stage IIA or greater^17^; and severe intraventricular hemorrhage, defined as grade 3 or 4.^18^ Bronchopulmonary dysplasia (BPD) was defined as supplemental oxygen use at 36 weeks’ postmenstrual age. The outcome of BPD was also subclassified using Jensen criteria for highest mode of respiratory support at 36 weeks’ postmenstrual age, with any BPD (grades 1, 2, or 3) or with a composite of grade 2 or grade 3 BPD (infants requiring positive pressure support).^19^

### Statistical Analysis

Based on an estimated sample size of 200 infants who underwent catheter closure and 375 infants who underwent surgical ligation during the 5-year study period, a 2-tailed α of .05, and 80% power, we projected detection of a difference in 6.5 total ventilator days or more between groups. Changes in rates of PDA treatment across the study period were assessed with the Cochrane-Armitage test for trend. Perinatal and neonatal characteristics, pulmonary characteristics, types of pharmacologic therapy used prior to definitive closure, and other neonatal morbidities were compared between the PDA treatment groups of transcatheter closure and surgical ligation using the χ^2^ test for categorical variables and the *t* test or the Wilcoxon rank sum test for continuous variables after testing for normality. The primary outcome was total days of mechanical ventilation. Multivariable linear regression was used to compare total days of mechanical ventilation between the transcatheter closure and surgical ligation groups adjusted for confounding variables in the model, including center, birth year, gestational age, and age at PDA intervention. Adjustment by center and year was performed to account for center differences in approach to respiratory care and timing of extubation as well as center variation in experience and volume of transcatheter closure procedures over time. Additional adjustment for prior pharmacologic PDA treatment was included in models. Similar adjusted linear and logistic regression analyses were conducted for secondary outcomes, including days of positive pressure ventilation and length of hospital stay as well as for the categorical outcomes of BPD, death, diuretic use at discharge, and oxygen use at discharge. Kruskal-Wallis testing was used to analyze age at PDA intervention across the study period. Statistical analyses were conducted from October 2021 to February 2024 using SAS software version 9.4 (SAS Institute). A 2-sided value of *P* < .05 was considered statistically significant.

## Results

**Figure 1.**
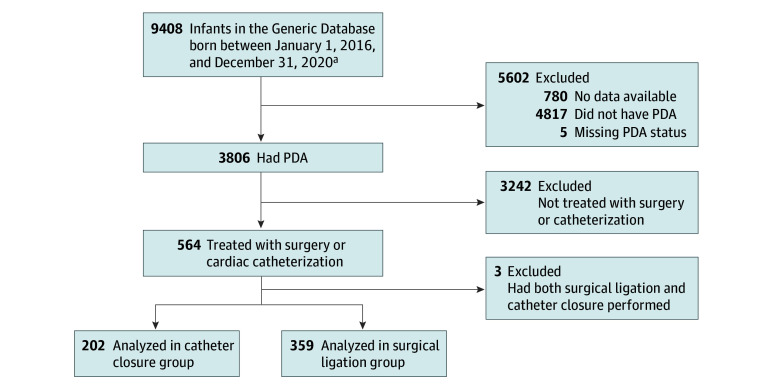
Study Population Flow Diagram PDA represents patent ductus arteriosus. ^a^Excludes infants who were outborn or had congenital malformations or syndromes.

During the 5-year study period, 3806 extremely preterm infants born at NRN centers had a PDA diagnosis. Of them, 202 infants (5.3%) underwent transcatheter PDA closure (median [IQR] gestational age, 25.4 [24.1-27.1] weeks; 114 [56%] female and 88 [44%] male) and 359 infants (9.4%) underwent surgical ligation (median [IQR] gestational age, 24.9 [24.0-25.9] weeks; 187 [52%] female and 172 [48%] were male) (Figure 1). Of 199 mothers whose infants underwent transcatheter PDA closure, 74 (37%) were Black and 113 (57%) were White. Of 346 mothers whose infants underwent surgical ligation, 143 (41%) were Black and 170 (49%) were White. Infants receiving surgical ligation and infants with transcatheter PDA closure had comparable unadjusted respiratory outcomes, including median (IQR) total days of mechanical ventilation (46 [30-68] days vs 46 [21-73] days), positive pressure support (80 [63-106] days vs 86 [66-117] days), and frequency of home oxygen use (185 [60%] vs 99 [55%]) (Table 1). The numbers of ventilator or oxygen use days after PDA intervention were not available from the GDB. In unadjusted analyses, more infants in the transcatheter PDA closure group had grade 2 or 3 BPD (150 of 201 [75%] vs 218 of 345 [63%]); however, more infants in the surgical ligation group required diuretics at discharge (119 of 306 [39%] vs 50 of 181 [28%]). Of note, after adjustment for center, birth year, gestational age, age at PDA closure, and prior pharmacologic treatment, none of the respiratory outcomes were different between groups (eg, adjusted median difference for days on mechanical ventilation, −2.65 [95% CI, −8.36 to 3.07]; *P* = .36). Moreover, length of hospital stay, BPD, death prior to discharge, and composite of death or BPD were not different between groups.

**Table 1.** Regression Models for Respiratory Outcomes Between Catheter Closure and Surgical Ligation

Outcome	Catheter closure		Surgical ligation		Unadjusted *P* value^a^	Model 1: base model		Model 2: includes prior pharmacologic treatment	
	Total No. of infants	No. (%) of infants	Total No. of infants	No. (%) of infants					
						Adjusted OR (95% CI)^b^	Adjusted *P* value^b^	Adjusted OR (95% CI)^c^	Adjusted *P* value^c^
Categorical outcome									
BPD^d^									
Supplemental oxygen	201	165 (82)	348	287 (82)	.91	1.20 (0.60 to 2.40)	.61	1.25 (0.62 to 2.52)	.53
Grade 1, 2, or 3	201	187 (93)	346	318 (92)	.63	0.71 (0.24 to 2.09)	.54	0.77 (0.26 to 2.30)	.64
Grade 2 or 3	201	150 (75)	345	218 (63)	.008	1.06 (0.59 to 1.91)	.86	1.05 (0.58 to 1.90)	.86
Death prior to discharge	200	6 (3)	351	22 (6)	.09	0.42 (0.12 to 1.46)	.17	0.40 (0.11 to 1.42)	.16
Death prior to discharge or supplemental oxygen	202	166 (82)	358	298 (83)	.75	1.13 (0.56 to 2.26)	.74	1.19 (0.59 to 2.40)	.63
Death prior to discharge or BPD grade 1, 2, or 3	202	188 (93)	356	329 (92)	.78	0.66 (0.22 to 1.95)	.45	0.72 (0.24 to 2.16)	.55
Death prior to discharge or BPD grade 2 or 3	202	151 (75)	354	229 (65)	.01	1.03 (0.57 to 1.85)	.93	1.02 (0.57 to 1.84)	.94
Home oxygen at discharge	181	99 (55)	306	185 (60)	.21	0.71 (0.39 to 1.29)	.26	0.72 (0.39 to 1.31)	.28
Diuretics at discharge	181	50 (28)	306	119 (39)	.01	0.92 (0.49 to 1.70)	.78	0.92 (0.50 to 1.71)	.80
Continuous outcomes among survivors									
Days of mechanical ventilation	194	46 (21-73)^e^	333	46 (30-68)^e^	.69	−3.07 (−9.36 to 3.22)^f^	.34	−2.65 (−8.36 to 3.07)^f^	.36
Days of positive pressure respiratory support	194	86 (66-117)^e^	333	80 (63-106)^e^	.12	−4.29 (−7.80 to −0.79)^f^	.02	−3.87 (−8.76 to 1.02)^f^	.12
Days in hospital	191	135 (112-176)^e^	326	136 (114-172)^e^	.85	−5.84 (−17.37 to 5.68)^f^	.32	−4.64 (−16.27 to 6.99)^f^	.43

Abbreviations: BPD, bronchopulmonary dysplasia; OR, odds ratio.

^a^ Differences in categorical outcomes were tested for by χ^2^ test; mean differences by *t* test.

^b^ For model 1, the adjusted median difference (95% CI), odds ratio (95% CI), and associated *P* value for each outcome between catheter closure and surgical ligation were obtained from quantile linear and logistic regression models, respectively, that adjusted for center, birth year, gestational age, and age at patent ductus arteriosus closure.

^c^ Model 2 is the same as model 1 but further adjusted for prior pharmacologic treatment.

^d^ BPD grades 1, 2, and 3 classified using Jensen criteria for highest mode of respiratory support at 36 weeks' postmenstrual age.^19^

^e^ Values are median (IQR).

^f^ Values are median difference (95% CI).

**Figure 2.**
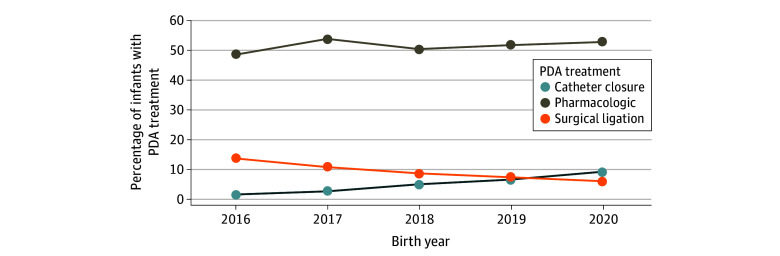
Patent Ductus Arteriosus (PDA) Treatment by Birth Year

Trends in PDA treatment across the study period are shown in Figure 2. The frequency of ever receiving pharmacologic PDA treatment remained relatively constant (*P* = .44 for trend); however, while surgical ligation rates declined (*P* < .001 for trend), rates of transcatheter PDA closure increased (*P* < .001 for trend). Notably, during the most recent study year (2020), of 773 infants with a PDA diagnosis, a higher percentage of infants had transcatheter PDA closure (73 [9%]) compared with surgical ligation (49 [6%]). The annual median (range) percentage of infants who were diagnosed with a PDA also remained unchanged at 44.6% (42.4%-45.3%) (*P* = .51) across the study period.

Maternal and perinatal characteristics were not different between transcatheter closure and surgical ligation groups (Table 2), although median (IQR) gestational age (25.4 [24.1-27.1] weeks vs 24.9 [24.0-25.9] weeks; *P* < .001) and weight at birth (740 [610-880] g vs 685 [585-810] g; *P* = .004) were higher in the transcatheter closure group. PDA treatment characteristics are shown in Table 3. Both postnatal age (mean [SD], 58.7 [28.4] vs 33.6 [16.7] days; *P* < .001) and postmenstrual age (mean [SD], 34.1 [4.5] vs 29.8 [2.8] weeks; *P* < .001) were greater in the transcatheter closure compared with surgical ligation groups. More infants in the surgical ligation group received prophylactic indomethacin or ever received treatments to modulate hemodynamic significance, such as indomethacin, acetaminophen, or more than 1 type of pharmacologic PDA treatment. Additional neonatal morbidities were compared between groups (Table 3). Infants in the transcatheter closure group were less likely to have had a pneumothorax at any time during their hospital course, but more commonly had preintervention NEC (27 of 201 [13%] vs 12 of 354 [3%]). For infants who developed culture-positive sepsis, the majority of events occurred prior to PDA closure, but preintervention sepsis rates (45 of 201 [22%] vs 75 of 346 [22%]) and overall sepsis rates did not differ between groups. Other pulmonary characteristics were not different between groups. Two infants died within 7 days of PDA intervention. One infant who underwent transcatheter PDA closure died after culture-positive sepsis, and 1 infant died of respiratory failure following PDA ligation.

**Table 2.** Perinatal Characteristics

Characteristic	Catheter closure (n = 202)		Surgical ligation (n = 359)		*P* value^a^
	Participants, total No.	Participants, No. (%)	Participants, total No.	Participants, No. (%)	
Maternal					
Race					
Black	199	74 (37)	346	143 (41)	.15
White	199	113 (57)	346	170 (49)	
Other^b^	199	12 (6)	346	33 (10)	
Hispanic ethnicity	202	37 (18)	355	67 (19)	.87
Chorioamnionitis^c^	202	92 (46)	359	180 (50)	.30
Preeclampsia	202	64 (32)	359	90 (25)	.09
Antenatal steroid	202	188 (93)	359	338 (94)	.61
Cesarean delivery	202	135 (67)	359	244 (68)	.78
Neonatal					
Gestational age, median (IQR), wk	202	25.4 (24.1-27.1)	359	24.9 (24.0-25.9)	<.001
Birth weight, median (IQR), g	202	740 (610-880)	359	685 (585-810)	.004
Sex				NA	NA
Female	202	114 (56)	359	187 (52)	.32
Male	202	88 (44)	359	172 (48)	
Small for gestational age	202	23 (11)	359	34 (9)	.47
5-min Apgar score, median (IQR)^d^	202	7 (5-8)	358	7 (5-8)	.32

Abbreviation: NA, not applicable.

^a^ Differences in categorical variables were tested for by χ^2^ test; differences in continuous variables, by Wilcoxon rank-sum test.

^b^ Includes American Indian or Alaska Native, Asian, Native Hawaiian or Other Pacific Islander, or more than 1 race.

^c^ Clinical or histologic chorioamnionitis.

^d^ Apgar score ranges from 1 to 10, with higher scores indicating better health.

**Table 3.** Patent Ductus Arteriosus Treatment Characteristics

Characteristic	Catheter closure (n = 202)		Surgical ligation (n = 359)		*P* value^a^
	Infants, total No.	Infants, No. (%)	Infants, total No.	Infants, No. (%)	
Age at intervention, mean (SD), d	199	58.7 (28.4)	322	33.6 (16.7)	<.001
Postmenstrual age at intervention, mean (SD), wk	199	34.1 (4.5)	322	29.8 (2.8)	<.001
Previous pharmacologic therapies to treat PDA					
Indomethacin	202	59 (29)	357	145 (41)	.009
Ibuprofen	202	62 (31)	359	127 (35)	.26
Acetaminophen	202	46 (23)	359	119 (33)	.01
At least 1 pharmacologic treatment	202	139 (69)	359	287 (80)	.004
>1 Pharmacologic treatment	202	35 (17)	359	150 (42)	<.001
Prophylactic indomethacin	201	15 (7)	357	94 (26)	<.001
Other morbidities					
NEC	202	32 (16)	358	33 (9)	.02
Surgical NEC	202	14 (7)	358	16 (4)	.21
NEC prior to PDA closure^b^	31	27 (87)	29	12 (41)	<.001
Spontaneous gastrointestinal tract perforation	202	12 (6)	358	33 (9)	.17
Severe IVH (grade 3 or 4)^c^	202	48 (24)	359	69 (19)	.20
Culture positive sepsis	202	54 (27)	359	123 (34)	.07
Sepsis prior to PDA closure^d^	53	45 (85)	110	75 (68)	.02
Pulmonary characteristics					
Pneumothorax	202	10 (5)	359	40 (11)	.01
Pulmonary interstitial emphysema	202	33 (16)	359	76 (21)	.16
Pulmonary hemorrhage	202	16 (8)	359	39 (11)	.26
Use of inhaled nitric oxide	202	43 (21)	359	58 (16)	.13
Received surfactant	202	189 (94)	359	341 (95)	.48
Steroid use for BPD	201	89 (44)	348	170 (49)	.30

Abbreviations: BPD, bronchopulmonary dysplasia; IVH, intraventricular hemorrhage; NEC, necrotizing enterocolitis; PDA, patent ductus arteriosus.

^a^ Differences in categorical variables were tested for by χ^2^ test; differences in continuous variables, by *t* test.

^b^ Percentage of all infants with NEC in each group.

^c^ Grade 3 or 4 IVH defined by Papile classification.^18^

^d^ Percentage of all infants with culture-positive sepsis in each group.

Subgroup analyses explored the association of center experience with transcatheter PDA closure and age of PDA closure on outcomes. Restricting analyses to centers that performed more than 20 catheter procedures during the study period did not alter the rate of respiratory outcomes between groups. Among infants with late PDA closure (>30 days after birth), 171 underwent transcatheter closure and 165 had surgical ligation. For those with early PDA closure (≤30 days after birth), 28 infants underwent transcatheter closure and 159 had surgical ligation. None of the outcome measures were significantly different between groups in either early or late closure strata. Regression coefficients in a model adjusting for age at PDA closure demonstrated a significant association of age at closure with the outcomes of grade 2 or 3 BPD, days of positive pressure respiratory support, and days in hospital (eTable 1 in Supplement 1). Age at transcatheter PDA closure decreased over time with a mean (SD) age of 90 (24) days in 2016 compared with a mean (SD) age of 53 (30) days in 2020 (*P* < .001). The mean (SD) age at surgical PDA ligation did not change across the study period, ranging from 30 (14) days to 35 (19) days (*P* = .65) (eTable 2 in Supplement 1). Grade 2 or 3 BPD rates did not significantly change across the study period.

## Discussion

In a retrospective analysis of a multicenter neonatal database during a 5-year period, this cohort study found that transcatheter PDA closure rates in the preterm infant surpassed surgical ligation rates for definitive PDA closure. Respiratory outcomes did not differ for infants treated with transcatheter PDA closure compared with surgical ligation. However, innate differences between groups existed. Lower frequency of prior pharmacologic PDA treatment, higher rate of preintervention NEC, and older chronologic and postmenstrual age at definitive PDA closure by the transcatheter route may be confounding factors. To adequately address the critical question of whether the benefits of transcatheter PDA closure outweigh the risks, randomized clinical trials comparing transcatheter PDA closure with alternative treatment strategies are needed.

The timing of definitive PDA closure has substantial implications given differences between groups. Infants who underwent PDA catheter closure were older than those who underwent surgical ligation, hence creating a possible imbalance in burden of PDA exposure. This confound was notable as adjustment for age at definitive PDA closure rendered all respiratory outcomes no longer significantly different between groups. Exposure to a hemodynamically significant PDA for a longer period of time may affect respiratory outcomes.^20,21,22,23,24^ In a study of infants born at less than 28 weeks’ gestation, those with a moderate to large PDA for 7 to 13 days had increased odds of BPD or death compared with those with a PDA for less than 7 days, although further exposure of 14 or more days was not associated with worse outcomes.^21^ In a single-center retrospective, case-control study of extremely preterm infants, longer duration of ductal patency was also associated with the development of BPD-associated pulmonary hypertension and mortality.^23^ In contrast, in both an observational study and a secondary analysis of a multicenter randomized clinical trial, prolonged PDA shunts were associated with increased BPD, but only when infants required intubation for 10 or more days.^22,24^ While difficult to ascertain the precise effect of longer exposure to a hemodynamically significant PDA, any influence of catheter closure on respiratory outcomes may be obscured by longer exposure to an open ductus. In the current study, modeling shows that the age at PDA closure could explain a large portion of the variance in outcomes for grade 2 or 3 BPD, days of positive pressure ventilation, and days in the hospital. As evidenced by decreasing age at catheter intervention during the study period, transcatheter PDA closure will likely extend to a growing number of smaller and younger infants, particularly as technical experience grows. An ongoing multicenter clinical trial^25^ is currently investigating PDA catheter closure at less than 53 days after birth, which represents a much younger cohort compared with the mean age of 58.7 days in the current study cohort. If randomized clinical trials demonstrate that earlier intervention does have a greater effect on BPD reduction, it will be necessary to account for shunt burden (magnitude and duration) and age at PDA closure in future studies.

An additional difference between groups was the increased rate of NEC prior to catheter closure compared with the surgical ligation group. Patient selection for a catheter intervention may have been influenced by the presence of this comorbidity. In addition, whether this higher rate of NEC is associated with shunt burden with more prolonged exposure to a greater magnitude PDA shunt in the transcatheter group is challenging to determine and requires prospective evaluation. Moderate to severe BPD may also occur in infants with a history of surgical NEC,^26^ possibly related to prolonged mechanical ventilatory needs, delayed enteral nutrition, and a persistent proinflammatory state. Thus, the increased occurrence of NEC may have obscured any benefit of catheter PDA closure on respiratory outcomes.

Other neonatal studies suggest improved respiratory outcomes with transcatheter PDA closure compared with surgical ligation, but these studies were single center or limited in size.^27,28,29,30,31,32,33^ The largest of these retrospective studies, which analyzed 64 infants who underwent transcatheter closure matched to 83 infants who underwent surgical ligation from 3 centers, concluded that the median duration of mechanical ventilation was shorter after transcatheter closure compared with surgical ligation (3 vs 5 days; *P* = .03), although there was no difference in total days of positive pressure ventilation or oxygen use at discharge.^27^ Most studies and a recent meta-analysis have found no difference in BPD between groups.^11,28,31,33,34,35^ We speculate that patient factors, including stability for transport to the catheterization laboratory at most sites, may bias toward the selection of a more stable population with fewer ventilator days. In contrast, a longer period of mechanical ventilation in the surgical ligation group may occur due to PLCS, increased need for sedation, or postoperative complications after manipulation of the left lung, such as atelectasis or pneumothorax.

The only other multicenter database describing respiratory outcomes in transcatheter PDA closure compared with surgical ligation, to our knowledge, comes from the Pediatric Health Information System administrative database in patients younger than 1 year.^36^ That analysis found decreased use of mechanical ventilation in the transcatheter closure group with an adjusted odds ratio of 0.3 (95% CI, 0.19-0.56). However, the study included both preterm and full-term infants and PDA closure beyond the neonatal period. When restricted to the limited neonatal population, there were no differences in BPD between groups.^36^ A separate descriptive analysis from this database of very low-birth-weight infants younger than 32 weeks’ gestation also found no difference in extubation rates between groups (13% vs 11%) in those already intubated prior to PDA closure.^10^

### Limitations

While the present study allowed for comparison of outcomes after definitive PDA closure using a large, multicenter database, study limitations exist. In addition to being a retrospective analysis, there was not a standardized echocardiography definition of PDA hemodynamic significance, which may create heterogeneity in the population. Specific echocardiographic parameters were not available from the database to compare size and hemodynamic significance of the PDA prior to the choice of intervention. Some infants may only have been surgical candidates and not eligible for transcatheter closure due to ductal size or configuration limitations. Furthermore, prior pharmacologic treatment was more common in the surgical ligation group, possibly resulting in incomplete ductal closure. Partial ductal closure could positively or negatively impact respiratory outcomes by either indicating a less hemodynamically significant shunt or greater severity of illness at the outset of definitive closure. Nonetheless, adjustment for prior pharmacologic treatment did not show differences in respiratory outcomes. Data were also unavailable regarding the mode of ventilation at the time of PDA closure or the number of days of mechanical ventilation after PDA closure; hence, total days of mechanical ventilation was chosen as the primary outcome and a more comprehensive assessment of respiratory status. However, this measure may not reflect potential respiratory benefits that may occur as a result of the specific PDA intervention.^27,28,29,30,31,36^ Pneumothorax is a known but rare potential complication of PDA surgical ligation,^37^ but the timing of pneumothorax was not available in this dataset. It remains unclear if pneumothorax occurred more frequently in the surgical ligation group as a result of the intervention or as a preintervention morbidity. Evidence obtained within the past 4 years highlight the potential association of prolonged PDA exposure and risk of pulmonary vascular disease or chronic pulmonary hypertension.^23,38,39^ Unfortunately, these data are not routinely collected in the NRN database. Finally, rates of PLCS, pulmonary vein stenosis, or feeding tube use at discharge as a proxy for aspiration were not available. As postnatal age at surgery is inversely proportional to the risk of PLCS due to left ventricular dysfunction, the earlier age in the surgical group may have increased the likelihood of postinterventional instability and prolonged mechanical ventilation. Prospective tracking of these outcomes may further inform considerations for optimal PDA management strategies.

## Conclusions

This cohort study found that respiratory outcomes did not differ between extremely preterm infants who underwent transcatheter PDA closure compared with surgical ligation, despite a longer duration of shunt exposure in the transcatheter closure group. Comparison of PDA closure at similar time points with standardized preintervention echocardiographic data collection is needed to address the knowledge gap of tolerable duration of PDA exposure and optimal timing of intervention on respiratory outcomes.

## Notes

Supplement 1. **eTable 1.** Regression Coefficients Adjusting for Age at PDA Closure **eTable 2.** Age at PDA Treatment Over Time

Supplement 2.
Nonauthor Collaborators

Supplement 3.
Data Sharing Statement

## References

[1] Bischoff AR, Jasani B, Sathanandam SK, Backes C, Weisz DE, McNamara PJ. Percutaneous closure of patent ductus arteriosus in infants 1.5 kg or less: a meta-analysis. J Pediatr. 2021;230:84-92.e14. doi:10.1016/j.jpeds.2020.10.035 33098843

[2] Backes CH, Rivera BK, Bridge JA, . Percutaneous patent ductus arteriosus (PDA) closure during infancy: a meta-analysis. Pediatrics. 2017;139(2):e20162927. doi:10.1542/peds.2016-2927 28087683

[3] Sathanandam SK, Gutfinger D, O’Brien L, . Amplatzer Piccolo Occluder clinical trial for percutaneous closure of the patent ductus arteriosus in patients ≥700 grams. Catheter Cardiovasc Interv. 2020;96(6):1266-1276. doi:10.1002/ccd.28973 32433821 PMC7754477

[4] Morray BH, Sathanandam SK, Forbes T, . 3-year follow-up of a prospective, multicenter study of the Amplatzer Piccolo™ Occluder for transcatheter patent ductus arteriosus closure in children ≥ 700 grams. J Perinatol. 2023;43(10):1238-1244. doi:10.1038/s41372-023-01741-1 37587183 PMC10541325

[5] Tabb C, Aggarwal S, Bajaj M, Natarajan G. Comparative effectiveness of surgical ligation and catheter closure of patent ductus arteriosus in preterm infants. Pediatr Cardiol. 2024; 45(7):1515-1523. doi:10.1007/s00246-023-03199-6 37316609

[6] Sathanandam S, Gutfinger D, Morray B, . Consensus guidelines for the prevention and management of periprocedural complications of transcatheter patent ductus arteriosus closure with the Amplatzer Piccolo Occluder in extremely low birth weight infants. Pediatr Cardiol. 2021;42(6):1258-1274. doi:10.1007/s00246-021-02665-3 34195869 PMC8292293

[7] O’Byrne ML, Millenson ME, Grady CB, . Trends in transcatheter and operative closure of patent ductus arteriosus in neonatal intensive care units: analysis of data from the Pediatric Health Information Systems Database. Am Heart J. 2019;217:121-130. doi:10.1016/j.ahj.2019.08.009 31654942 PMC6861695

[8] Kaluarachchi DC, Rysavy MA, Carper BA, . Secular trends in patent ductus arteriosus management in infants born preterm in the National Institute of Child Health and Human Development Neonatal Research Network. J Pediatr. 2024;266:113877. doi:10.1016/j.jpeds.2023.113877 38135028 PMC10922632

[9] Shah ZS, Clark RH, Patt HA, Backes CH Jr, Tolia VN. Trends in procedural closure of the patent ductus arteriosus among infants born at 22 to 30 weeks’ gestation. J Pediatr. 2023;263:113716. doi:10.1016/j.jpeds.2023.113716 37659585

[10] Lai KC, Richardson T, Berman D, . Current trends in invasive closure of patent ductus arteriosus in very low birth weight infants in United States children’s hospitals, 2016-2021. J Pediatr. 2023;263:113712. doi:10.1016/j.jpeds.2023.113712 37659587

[11] Leahy BF, Edwards EM, Ehret DEY, Soll RF, Yeager SB, Flyer JN. Transcatheter and surgical ductus arteriosus closure in very low birth weight infants: 2018-2022. Pediatrics. 2024;154(2):e2024065905. doi:10.1542/peds.2024-065905 39005106

[12] Kabra NS, Schmidt B, Roberts RS, Doyle LW, Papile L, Fanaroff A; Trial of Indomethacin Prophylaxis in Preterms Investigators. Neurosensory impairment after surgical closure of patent ductus arteriosus in extremely low birth weight infants: results from the Trial of Indomethacin Prophylaxis in Preterms. J Pediatr. 2007;150(3):229-234, 234.e1. doi:10.1016/j.jpeds.2006.11.039 17307535

[13] McNamara PJ, Stewart L, Shivananda SP, Stephens D, Sehgal A. Patent ductus arteriosus ligation is associated with impaired left ventricular systolic performance in premature infants weighing less than 1000 g. J Thorac Cardiovasc Surg. 2010;140(1):150-157. doi:10.1016/j.jtcvs.2010.01.011 20363478

[14] Chock VY, Ramamoorthy C, Van Meurs KP. Cerebral autoregulation in neonates with a hemodynamically significant patent ductus arteriosus. J Pediatr. 2012;160(6):936-942. doi:10.1016/j.jpeds.2011.11.054 22226574 PMC3335982

[15] Backes CH, Giesinger RE, Rivera BK, . Percutaneous closure of the patent ductus arteriosus in very low weight infants: considerations following US Food and Drug Administration approval of a novel device. J Pediatr. 2019;213:218-221. doi:10.1016/j.jpeds.2019.05.062 31255391

[16] Flanagin A, Frey T, Christiansen SL; AMA Manual of Style Committee. Updated guidance on the reporting of race and ethnicity in medical and science journals. JAMA. 2021;326(7):621-627. doi:10.1001/jama.2021.13304 34402850

[17] Bell MJ, Ternberg JL, Feigin RD, . Neonatal necrotizing enterocolitis: therapeutic decisions based upon clinical staging. Ann Surg. 1978;187(1):1-7. doi:10.1097/00000658-197801000-00001 413500 PMC1396409

[18] Papile LA, Burstein J, Burstein R, Koffler H. Incidence and evolution of subependymal and intraventricular hemorrhage: a study of infants with birth weights less than 1,500 gm. J Pediatr. 1978;92(4):529-534. doi:10.1016/S0022-3476(78)80282-0 305471

[19] Jensen EA, Dysart K, Gantz MG, . The diagnosis of bronchopulmonary dysplasia in very preterm infants: an evidence-based approach. Am J Respir Crit Care Med. 2019;200(6):751-759. doi:10.1164/rccm.201812-2348OC 30995069 PMC6775872

[20] Mirza H, Garcia J, McKinley G, . Duration of significant patent ductus arteriosus and bronchopulmonary dysplasia in extremely preterm infants. J Perinatol. 2019;39(12):1648-1655. doi:10.1038/s41372-019-0496-5 31554913

[21] Clyman RI, Hills NK, Liebowitz M, Johng S. Relationship between duration of infant exposure to a moderate-to-large patent ductus arteriosus shunt and the risk of developing bronchopulmonary dysplasia or death before 36 weeks. Am J Perinatol. 2020;37(2):216-223. doi:10.1055/s-0039-1697672 31600791 PMC9940607

[22] Clyman RI, Hills NK, Cambonie G, . Patent ductus arteriosus, tracheal ventilation, and the risk of bronchopulmonary dysplasia. Pediatr Res. 2022;91(3):652-658. doi:10.1038/s41390-021-01475-w 33790415 PMC8904244

[23] Gentle SJ, Travers CP, Clark M, Carlo WA, Ambalavanan N. Patent ductus arteriosus and development of bronchopulmonary dysplasia-associated pulmonary hypertension. Am J Respir Crit Care Med. 2023;207(7):921-928. doi:10.1164/rccm.202203-0570OC 36378949 PMC10111998

[24] Clyman RI, Hills NK. The effect of prolonged tracheal intubation on the association between patent ductus arteriosus and bronchopulmonary dysplasia (grades 2 and 3). J Perinatol. 2020;40(9):1358-1365. doi:10.1038/s41372-020-0718-x 32669644 PMC7442702

[25] Percutaneous Intervention Versus Observational Trial of Arterial Ductus in Low Weight Infants (PIVOTAL). ClinicalTrials.gov identifier: NCT05547165. Updated March 30, 2025. Accessed April 16, 2025. https://clinicaltrials.gov/study/NCT05547165?term=NCT05547165&rank=1

[26] Garg PM, Pippin M, Zhang M, . Clinical correlates of moderate-to-severe bronchopulmonary dysplasia in preterm infants following surgical necrotizing enterocolitis. Am J Perinatol. 2024;41(10):1348-1358. doi:10.1055/a-1904-9194 35858647 PMC10278056

[27] Regan W, Benbrik N, Sharma SR, . Improved ventilation in premature babies after transcatheter versus surgical closure of patent ductus arteriosus. Int J Cardiol. 2020;311:22-27. doi:10.1016/j.ijcard.2020.03.040 32253052

[28] Abu Hazeem AA, Gillespie MJ, Thun H, . Percutaneous closure of patent ductus arteriosus in small infants with significant lung disease may offer faster recovery of respiratory function when compared to surgical ligation. Catheter Cardiovasc Interv. 2013;82(4):526-533. doi:10.1002/ccd.25032 23723091

[29] Pamukcu O, Tuncay A, Narin N, . Patent ductus arteriosus closure in preterms less than 2kg: surgery versus transcatheter. Int J Cardiol. 2018;250:110-115. doi:10.1016/j.ijcard.2017.10.020 29017778

[30] Sathanandam S, Balduf K, Chilakala S, . Role of transcatheter patent ductus arteriosus closure in extremely low birth weight infants. Catheter Cardiovasc Interv. 2019;93(1):89-96. doi:10.1002/ccd.27808 30269408

[31] Rodríguez Ogando A, Planelles Asensio I, de la Blanca ARS, . Surgical ligation versus percutaneous closure of patent ductus arteriosus in very low-weight preterm infants: which are the real benefits of the percutaneous approach? Pediatr Cardiol. 2018;39(2):398-410. doi:10.1007/s00246-017-1768-5 29119215

[32] Wheeler CR, Gagner D, Stephens H, . Phenotyping respiratory decompensation following definitive closure of the patent ductus arteriosus in preterm infants. J Perinatol. 2022;42(5):649-654. doi:10.1038/s41372-021-01226-z 34650199

[33] Fernandez MC, Kase JS, Giamelli J, Reichlin A. Morbidity and neurodevelopmental outcomes at 2 years in preterm infants undergoing percutaneous transcatheter closure vs. surgical ligation of the PDA. J Perinatol. 2024;44(10):1454-1462. doi:10.1038/s41372-024-02019-w 38831120

[34] Wei YJ, Ju YT, Hsieh ML, Kan CD, Lin YC, Wang JN. Surgical ligation, not transcatheter closure, associated with a higher severity of bronchopulmonary dysplasia in extremely preterm infant intervened for patent ductus arteriosus. Pediatr Pulmonol. 2023;58(4):1221-1228. doi:10.1002/ppul.26325 36696083

[35] Melchior CDS, Neves GR, de Oliveira BL, . Percutaneous closure of patent ductus arteriosus versus surgical treatment in low-birth-weight preterms: a systematic review and meta-analysis. Cardiol Young. 2024;34(4):705-712. doi:10.1017/S1047951123004353 38329109

[36] Kuntz MT, Staffa SJ, Graham D, . Trend and outcomes for surgical versus transcatheter patent ductus arteriosus closure in neonates and infants at US children’s hospitals. J Am Heart Assoc. 2022;11(1):e022776. doi:10.1161/JAHA.121.022776 34970919 PMC9075185

[37] Ashfaq A, Rettig RL, Chong A, Sydorak R. Outcomes of patent ductus arteriosus ligation in very low birth weight premature infants: a retrospective cohort analysis. J Pediatr Surg. 2022;57(7):1201-1204. doi:10.1016/j.jpedsurg.2022.02.037 35450698

[38] Philip R, Waller BR, Chilakala S, . Hemodynamic and clinical consequences of early versus delayed closure of patent ductus arteriosus in extremely low birth weight infants. J Perinatol. 2021;41(1):100-108. doi:10.1038/s41372-020-00772-2 32792636

[39] Nawaytou H, Hills NK, Clyman RI. Patent ductus arteriosus and the risk of bronchopulmonary dysplasia-associated pulmonary hypertension. Pediatr Res. 2023;94(2):547-554. doi:10.1038/s41390-023-02522-4 36804505 PMC10403370

