# Supplementary material for: Respiratory Outcomes After Transcatheter vs Surgical Patent Ductus Arteriosus Closure in Preterm Infants
Source: JAMA Netw Open. 2025 Jun 3;8(6):e2513366. doi: 10.1001/jamanetworkopen.2025.13366 (PMC12134952; doi:10.1001/jamanetworkopen.2025.13366)
Supplement: Supplement 1. — eTable 1. Regression Coefficients Adjusting for Age at PDA Closure eTable 2. Age at PDA Treatment Over Time [file jamanetwopen-e2513366-s001.pdf]

Supplementary Online Content

Chock VY, Bhombal S, Davis AS, et al. Respiratory outcomes after transcatheter vs surgical patent ductus arteriosus closure in preterm infants. *JAMA Netw Open*. 2025;8(6):e2513366. doi:10.1001/jamanetworkopen.2025.13366

eTable 1. Regression Coefficients Adjusting for Age at PDA Closure
eTable 2. Age at PDA Treatment Over Time

This supplementary material has been provided by the authors to give readers additional information about their work.

© 2025 Chock VY et al. *JAMA Network Open*.

eTable 1. Regression Coefficients Adjusting for Age at PDA Closure

Categorical Outcomes	n	Catheter closure n=202	n	Surgical ligation n=359	Unadjusted P-value	β (SE)	Adjusted P-value
Days of mechanical ventilation, median (IQR)	194	46.5 (21, 73)	333	45 (30, 68)	0.69	0.1061 (0.0655)	0.11
Bronchopulmonary dysplasia							
Supplemental oxygen	201	165 (82)	348	287 (82)	0.91	-0.0005 (0.0057)	0.93
BPD grade 1, 2 or 3	201	187 (93)	346	318 (92)	0.63	-0.0007 (0.0082)	0.94
BPD grade 2 or 3	201	150 (75)	345	218 (63)	<0.01	0.0105 (0.0053)	0.05
Death prior to discharge	200	6 (3)	351	22 (6)	0.09	0.0091 (0.0100)	0.36
Death prior to discharge or supplemental oxygen	202	166 (82)	358	298 (83)	0.75	-0.0007 (0.0056)	0.90
Death prior to discharge or BPD grade 1, 2 or 3	202	188 (93)	356	329 (92)	0.78	-0.0003 (0.0083)	0.98
Death prior to discharge or BPD grade 2 or 3	202	151 (75)	354	229 (65)	0.01	0.0094 (0.0052)	0.07
Days on positive pressure respiratory support, median (IQR)	194	86 (66, 117)	333	80 (63,105)	0.12	0.2737 (0.0300)	<0.01
Home oxygen at discharge	181	99 (55)	306	185 (60)	0.21	-0.0032 (0.0051)	0.54
Diuretics at discharge	181	50 (28)	306	119 (39)	0.01	0.0026 (0.0051)	0.61
Days in hospital, median (IQR)	191	135 (112, 176)	326	135.5 (114, 171)	0.85	0.2691 (0.0955)	0.01

© 2025 Chock VY et al. *JAMA Network Open*.

eTable 2. Age at PDA Treatment Over Time

Treatment	Age at intervention by year, mean days (SD)					p-value
	2016	2017	2018	2019	2020	
Catheter closure	89.5 (24.3)	68.4 (16.9)	56.8 (24.3)	56.4 (28.3)	52.7 (30.3)	<0.001
Surgical ligation	35.0 (19.4)	32.5 (13.7)	33.5 (15.0)	35.3 (18.9)	30.4 (14.4)	0.65

© 2025 Chock VY et al. *JAMA Network Open*.
