# Supplementary material for: Respiratory Outcomes After Transcatheter vs Surgical Patent Ductus Arteriosus Closure in Preterm Infants
Source: JAMA Netw Open. 2025 Jun 3;8(6):e2513366. doi: 10.1001/jamanetworkopen.2025.13366 (PMC12134952; doi:10.1001/jamanetworkopen.2025.13366)
Supplement: Supplement 2. — Nonauthor Collaborators [file jamanetwopen-e2513366-s002.pdf]

*First name, last name, and suffix (if applicable) are required and will appear in PubMed.

***Group Name(s):** Eunice Kennedy Shriver National Institute of Child Health and Human Development Neonatal Research Network

***First Name and Middle Initial(s)**	***Last Name**	***Suffix (eg, Jr, III)**	Academic Degrees	Institution	Location (city, state/province, country)	Role or Contribution, eg, chair, principal investigator	Group (if more than 1 Group listed in the byline) and/or Subgroup (eg, Steering Committee)
Richard A.	Polin		MD	College of Physicians and Surgeons, Columbia University	New York, NY	NRN Steering Committee Chair	
Abbot R.	Laptook		MD	Alpert Medical School of Brown University and Women & Infants Hospital of Rhode Island	Providence, RI	Non-Author Contributor	
Martin	Keszler		MD	Alpert Medical School of Brown University and Women & Infants Hospital of Rhode Island	Providence, RI	Non-Author Contributor	
Angelita M.	Hensman		PhD RNC-NIC	Alpert Medical School of Brown University and Women & Infants Hospital of Rhode Island	Providence, RI	Non-Author Contributor	
Lucille	St. Pierre		BS	Alpert Medical School of Brown University and Women & Infants Hospital of Rhode Island	Providence, RI	Non-Author Contributor	
Elisa	Vieira		BSN RN	Alpert Medical School of Brown University and Women & Infants Hospital of Rhode Island	Providence, RI	Non-Author Contributor	
Anna Marie	Hibbs		MD	Case Western Reserve University, Rainbow Babies & Children's Hospital	Cleveland, OH	Non-Author Contributor	
Michele C.	Walsh		MD MS	Case Western Reserve University, Rainbow Babies & Children's Hospital	Cleveland, OH	Non-Author Contributor	
Nancy S.	Newman		RN	Case Western Reserve University, Rainbow Babies & Children's Hospital	Cleveland, OH	Non-Author Contributor	
Bonnie S.	Siner		RN	Case Western Reserve University, Rainbow Babies & Children's Hospital	Cleveland, OH	Non-Author Contributor	

*First name, last name, and suffix (if applicable) are required and will appear in PubMed.

***First Name and Middle Initial(s)**	***Last Name**	***Suffix (eg, Jr, III)**	Academic Degrees	Institution	Location (city, state/province, country)	Role or Contribution, eg, chair, principal investigator	Group (if more than 1 Group listed in the byline) and/or Subgroup (eg, Steering Committee)
Angelia	Williams			Case Western Reserve University, Rainbow Babies & Children's Hospital	Cleveland, OH	Non-Author Contributor	
William E.	Truog		MD	Children's Mercy Hospital, University of Missouri Kansas City School of Medicine and Kansas University Medical Center	Kansas City, MO	Non-Author Contributor	
Eugenia K.	Pallotto		MD MSCE	Children's Mercy Hospital, University of Missouri Kansas City School of Medicine and Kansas University Medical Center	Kansas City, MO	Non-Author Contributor	
Howard W.	Kilbride		MD	Children's Mercy Hospital, University of Missouri Kansas City School of Medicine and Kansas University Medical Center	Kansas City, MO	Non-Author Contributor	
Prabhu S.	Parimi		MD	Children's Mercy Hospital, University of Missouri Kansas City School of Medicine and Kansas University Medical Center	Kansas City, MO	Non-Author Contributor	
Cheri	Gauldin		RN BSN CCRC	Children's Mercy Hospital, University of Missouri Kansas City School of Medicine and Kansas University Medical Center	Kansas City, MO	Non-Author Contributor	
Lisa	Gaetano		RN MSN	Children's Mercy Hospital, University of Missouri Kansas City School of Medicine and Kansas University Medical Center	Kansas City, MO	Non-Author Contributor	
Anne	Holmes		RN MSN MBA-HCM CCRC	Children's Mercy Hospital, University of Missouri Kansas City School of Medicine and Kansas University Medical Center	Kansas City, MO	Non-Author Contributor	

Supplemental Online Content: Nonauthor Collaborators
*First name, last name, and suffix (if applicable) are required and will appear in PubMed.

***First Name and Middle Initial(s)**	***Last Name**	***Suffix (eg, Jr, III)**	Academic Degrees	Institution	Location (city, state/province, country)	Role or Contribution, eg, chair, principal investigator	Group (if more than 1 Group listed in the byline) and/or Subgroup (eg, Steering Committee)
Allison	Scott		BSN RNC-NIC	Children's Mercy Hospital, University of Missouri Kansas City School of Medicine and Kansas University Medical Center	Kansas City, MO	Non-Author Contributor	
Brenda B.	Poindexter		MD MS	Cincinnati Children's Hospital Medical Center, University Hospital, and Good Samaritan Hospital	Cincinnati, OH	Non-Author Contributor	
Stephanie L.	Merhar		MD MS	Cincinnati Children's Hospital Medical Center, University Hospital, and Good Samaritan Hospital	Cincinnati, OH	Non-Author Contributor	
Kurt	Schibler		MD	Cincinnati Children's Hospital Medical Center, University Hospital, and Good Samaritan Hospital	Cincinnati, OH	Non-Author Contributor	
Cathy	Grisby		BSN CCRC	Cincinnati Children's Hospital Medical Center, University Hospital, and Good Samaritan Hospital	Cincinnati, OH	Non-Author Contributor	
Traci	Beiersdorfer		RN BSN	Cincinnati Children's Hospital Medical Center, University Hospital, and Good Samaritan Hospital	Cincinnati, OH	Non-Author Contributor	
Kristin	Kirker		CRC	Cincinnati Children's Hospital Medical Center, University Hospital, and Good Samaritan Hospital	Cincinnati, OH	Non-Author Contributor	

Supplemental Online Content: Nonauthor Collaborators
*First name, last name, and suffix (if applicable) are required and will appear in PubMed.

***First Name and Middle Initial(s)**	***Last Name**	***Suffix (eg, Jr, III)**	Academic Degrees	Institution	Location (city, state/province, country)	Role or Contribution, eg, chair, principal investigator	Group (if more than 1 Group listed in the byline) and/or Subgroup (eg, Steering Committee)
David	Russell		JD	Cincinnati Children's Hospital Medical Center, University Hospital, and Good Samaritan Hospital	Cincinnati, OH	Non-Author Contributor	
Julia	Thompson		RN BSN	Cincinnati Children's Hospital Medical Center, University Hospital, and Good Samaritan Hospital	Cincinnati, OH	Non-Author Contributor	
C. Michael	Cotten		MD MHS	Duke University School of Medicine, University Hospital, University of North Carolina, Duke Regional Hospital, WakeMed Health & Hospitals, and Maynard Children's Hospital at East Caroline University Health	Durham, Chapel Hill, Raleigh, and Greenville, NC	Non-Author Contributor	
Ronald N.	Goldberg		MD	Duke University School of Medicine, University Hospital, University of North Carolina, Duke Regional Hospital, WakeMed Health & Hospitals, and Maynard Children's Hospital at East Caroline University Health	Durham, Chapel Hill, Raleigh, and Greenville, NC	Non-Author Contributor	
Joanne	Finkle		RN JD	Duke University School of Medicine, University Hospital, University of North Carolina, Duke Regional Hospital, WakeMed Health & Hospitals, and Maynard Children's Hospital at East Caroline University Health	Durham, Chapel Hill, Raleigh, and Greenville, NC	Non-Author Contributor	

Supplemental Online Content: Nonauthor Collaborators
*First name, last name, and suffix (if applicable) are required and will appear in PubMed.

***First Name and Middle Initial(s)**	***Last Name**	***Suffix (eg, Jr, III)**	Academic Degrees	Institution	Location (city, state/province, country)	Role or Contribution, eg, chair, principal investigator	Group (if more than 1 Group listed in the byline) and/or Subgroup (eg, Steering Committee)
Kimberley A.	Fisher		PhD FNP-BC IBCLC	Duke University School of Medicine, University Hospital, University of North Carolina, Duke Regional Hospital, WakeMed Health & Hospitals, and Maynard Children's Hospital at East Caroline University Health	Durham, Chapel Hill, Raleigh, and Greenville, NC	Non-Author Contributor	
Carl L.	Bose		MD	Duke University School of Medicine, University Hospital, University of North Carolina, Duke Regional Hospital, WakeMed Health & Hospitals, and Maynard Children's Hospital at East Caroline University Health	Durham, Chapel Hill, Raleigh, and Greenville, NC	Non-Author Contributor	
Janice	Bernhardt		MS RN	Duke University School of Medicine, University Hospital, University of North Carolina, Duke Regional Hospital, WakeMed Health & Hospitals, and Maynard Children's Hospital at East Caroline University Health	Durham, Chapel Hill, Raleigh, and Greenville, NC	Non-Author Contributor	
Gennie	Bose		RN	Duke University School of Medicine, University Hospital, University of North Carolina, Duke Regional Hospital, WakeMed Health & Hospitals, and Maynard Children's Hospital at East Caroline University Health	Durham, Chapel Hill, Raleigh, and Greenville, NC	Non-Author Contributor	

Supplemental Online Content: Nonauthor Collaborators
*First name, last name, and suffix (if applicable) are required and will appear in PubMed.

***First Name and Middle Initial(s)**	***Last Name**	***Suffix (eg, Jr, III)**	Academic Degrees	Institution	Location (city, state/province, country)	Role or Contribution, eg, chair, principal investigator	Group (if more than 1 Group listed in the byline) and/or Subgroup (eg, Steering Committee)
Cindy	Clark		RN	Duke University School of Medicine, University Hospital, University of North Carolina, Duke Regional Hospital, WakeMed Health & Hospitals, and Maynard Children's Hospital at East Caroline University Health	Durham, Chapel Hill, Raleigh, and Greenville, NC	Non-Author Contributor	
Jennifer	Talbert		MS RN BSN RDH	Duke University School of Medicine, University Hospital, University of North Carolina, Duke Regional Hospital, WakeMed Health & Hospitals, and Maynard Children's Hospital at East Caroline University Health	Durham, Chapel Hill, Raleigh, and Greenville, NC	Non-Author Contributor	
Stephen D.	Kicklighter		MD	Duke University School of Medicine, University Hospital, University of North Carolina, Duke Regional Hospital, WakeMed Health & Hospitals, and Maynard Children's Hospital at East Caroline University Health	Durham, Chapel Hill, Raleigh, and Greenville, NC	Non-Author Contributor	
Ginger	Rhodes-Ryan		ARNP MSN, NNP-BC	Duke University School of Medicine, University Hospital, University of North Carolina, Duke Regional Hospital, WakeMed Health & Hospitals, and Maynard Children's Hospital at East Caroline University Health	Durham, Chapel Hill, Raleigh, and Greenville, NC	Non-Author Contributor	

Supplemental Online Content: Nonauthor Collaborators
*First name, last name, and suffix (if applicable) are required and will appear in PubMed.

***First Name and Middle Initial(s)**	***Last Name**	***Suffix (eg, Jr, III)**	Academic Degrees	Institution	Location (city, state/province, country)	Role or Contribution, eg, chair, principal investigator	Group (if more than 1 Group listed in the byline) and/or Subgroup (eg, Steering Committee)
Donna	White		RN-BC, BSN	Duke University School of Medicine, University Hospital, University of North Carolina, Duke Regional Hospital, WakeMed Health & Hospitals, and Maynard Children's Hospital at East Caroline University Health	Durham, Chapel Hill, Raleigh, and Greenville, NC	Non-Author Contributor	
Ryan	Moore		MD	Duke University School of Medicine, University Hospital, University of North Carolina, Duke Regional Hospital, WakeMed Health & Hospitals, and Maynard Children's Hospital at East Caroline University Health	Durham, Chapel Hill, Raleigh, and Greenville, NC	Non-Author Contributor	
Kelly	Bear		MD	Duke University School of Medicine, University Hospital, University of North Carolina, Duke Regional Hospital, WakeMed Health & Hospitals, and Maynard Children's Hospital at East Caroline University Health	Durham, Chapel Hill, Raleigh, and Greenville, NC	Non-Author Contributor	
Sherry	Moseley		RN	Duke University School of Medicine, University Hospital, University of North Carolina, Duke Regional Hospital, WakeMed Health & Hospitals, and Maynard Children's Hospital at East Caroline University Health	Durham, Chapel Hill, Raleigh, and Greenville, NC	Non-Author Contributor	

Supplemental Online Content: Nonauthor Collaborators
*First name, last name, and suffix (if applicable) are required and will appear in PubMed.

***First Name and Middle Initial(s)**	***Last Name**	***Suffix (eg, Jr, III)**	Academic Degrees	Institution	Location (city, state/province, country)	Role or Contribution, eg, chair, principal investigator	Group (if more than 1 Group listed in the byline) and/or Subgroup (eg, Steering Committee)
Vicki	Bergstedt		RN	Duke University School of Medicine, University Hospital, University of North Carolina, Duke Regional Hospital, WakeMed Health & Hospitals, and Maynard Children's Hospital at East Caroline University Health	Durham, Chapel Hill, Raleigh, and Greenville, NC	Non-Author Contributor	
Ravi M.	Patel		MD MSc	Emory University, Children's Healthcare of Atlanta, Grady Memorial Hospital, and Emory University Hospital Midtown	Atlanta, GA	Non-Author Contributor	
David P.	Carlton		MD	Emory University, Children's Healthcare of Atlanta, Grady Memorial Hospital, and Emory University Hospital Midtown	Atlanta, GA	Non-Author Contributor	
Yvonne	Loggins		RN	Emory University, Children's Healthcare of Atlanta, Grady Memorial Hospital, and Emory University Hospital Midtown	Atlanta, GA	Non-Author Contributor	
Diane I.	Bottcher		RN MSN	Emory University, Children's Healthcare of Atlanta, Grady Memorial Hospital, and Emory University Hospital Midtown	Atlanta, GA	Non-Author Contributor	
Colleen	Mackie		BS RT	Emory University, Children's Healthcare of Atlanta, Grady Memorial Hospital, and Emory University Hospital Midtown	Atlanta, GA	Non-Author Contributor	
Andrew A.	Bremer		MD PhD	*Eunice Kennedy Shriver* National Institute of Child Health and Human Development	Bethesda, MD	Non-Author Contributor	

Supplemental Online Content: Nonauthor Collaborators
*First name, last name, and suffix (if applicable) are required and will appear in PubMed.

***First Name and Middle Initial(s)**	***Last Name**	***Suffix (eg, Jr, III)**	Academic Degrees	Institution	Location (city, state/province, country)	Role or Contribution, eg, chair, principal investigator	Group (if more than 1 Group listed in the byline) and/or Subgroup (eg, Steering Committee)
Rosemary D.	Higgins		MD	*Eunice Kennedy Shriver* National Institute of Child Health and Human Development	Bethesda, MD	Non-Author Contributor	
Stephanie	Wilson Archer		MA	*Eunice Kennedy Shriver* National Institute of Child Health and Human Development	Bethesda, MD	Non-Author Contributor	
Gregory M.	Sokol		MD	Indiana University, Methodist Hospital, Riley Hospital for Children at Indiana University Health, and Eskenazi Health	Indianapolis, IN	Non-Author Contributor	
Dianne E.	Herron		RN CCRC	Indiana University, Methodist Hospital, Riley Hospital for Children at Indiana University Health, and Eskenazi Health	Indianapolis, IN	Non-Author Contributor	
Susan	Gunn		NNP CCRC	Indiana University, Methodist Hospital, Riley Hospital for Children at Indiana University Health, and Eskenazi Health	Indianapolis, IN	Non-Author Contributor	
Jeffery	Joyce		CCRC	Indiana University, Methodist Hospital, Riley Hospital for Children at Indiana University Health, and Eskenazi Health	Indianapolis, IN	Non-Author Contributor	
Jon E.	Tyson		MD MPH	McGovern Medical School at The University of Texas Health Science Center at Houston, Children's Memorial Hermann Hospital, and Memorial Hermann Southwest Hospital	Houston, TX	Non-Author Contributor	

Supplemental Online Content: Nonauthor Collaborators
*First name, last name, and suffix (if applicable) are required and will appear in PubMed.

***First Name and Middle Initial(s)**	***Last Name**	***Suffix (eg, Jr, III)**	Academic Degrees	Institution	Location (city, state/province, country)	Role or Contribution, eg, chair, principal investigator	Group (if more than 1 Group listed in the byline) and/or Subgroup (eg, Steering Committee)
Amir M.	Khan		MD	McGovern Medical School at The University of Texas Health Science Center at Houston, Children's Memorial Hermann Hospital, and Memorial Hermann Southwest Hospital	Houston, TX	Non-Author Contributor	
Kathleen A.	Kennedy		MD MPH	McGovern Medical School at The University of Texas Health Science Center at Houston, Children's Memorial Hermann Hospital, and Memorial Hermann Southwest Hospital	Houston, TX	Non-Author Contributor	
Elizabeth	Eason		MD	McGovern Medical School at The University of Texas Health Science Center at Houston, Children's Memorial Hermann Hospital, and Memorial Hermann Southwest Hospital	Houston, TX	Non-Author Contributor	
Donna J.	Hall		RN	McGovern Medical School at The University of Texas Health Science Center at Houston, Children's Memorial Hermann Hospital, and Memorial Hermann Southwest Hospital	Houston, TX	Non-Author Contributor	
Karen	Martin		RN	McGovern Medical School at The University of Texas Health Science Center at Houston, Children's Memorial Hermann Hospital, and Memorial Hermann Southwest Hospital	Houston, TX	Non-Author Contributor	

Supplemental Online Content: Nonauthor Collaborators

*First name, last name, and suffix (if applicable) are required and will appear in PubMed.

***First Name and Middle Initial(s)**	***Last Name**	***Suffix (eg, Jr, III)**	Academic Degrees	Institution	Location (city, state/province, country)	Role or Contribution, eg, chair, principal investigator	Group (if more than 1 Group listed in the byline) and/or Subgroup (eg, Steering Committee)
Michelle	White		BSN RNC-NIC	McGovern Medical School at The University of Texas Health Science Center at Houston, Children's Memorial Hermann Hospital, and Memorial Hermann Southwest Hospital	Houston, TX	Non-Author Contributor	
Georgia E.	McDavid		RN	McGovern Medical School at The University of Texas Health Science Center at Houston, Children's Memorial Hermann Hospital, and Memorial Hermann Southwest Hospital	Houston, TX	Non-Author Contributor	
Emily K.	Stephens		BSN RNC-NIC	McGovern Medical School at The University of Texas Health Science Center at Houston, Children's Memorial Hermann Hospital, and Memorial Hermann Southwest Hospital	Houston, TX	Non-Author Contributor	
Sharon L.	Wright		MT	McGovern Medical School at The University of Texas Health Science Center at Houston, Children's Memorial Hermann Hospital, and Memorial Hermann Southwest Hospital	Houston, TX	Non-Author Contributor	
Pablo J.	Sanchez		MD	Nationwide Children's Hospital, Abigail Wexner Research Institute at Nationwide Children's Hospital, Center for Perinatal Research, The Ohio State University Wexner Medical Center, and Riverside Methodist Hospital	Columbus, OH	Non-Author Contributor	

Supplemental Online Content: Nonauthor Collaborators
*First name, last name, and suffix (if applicable) are required and will appear in PubMed.

***First Name and Middle Initial(s)**	***Last Name**	***Suffix (eg, Jr, III)**	Academic Degrees	Institution	Location (city, state/province, country)	Role or Contribution, eg, chair, principal investigator	Group (if more than 1 Group listed in the byline) and/or Subgroup (eg, Steering Committee)
Leif D.	Nelin		MD	Nationwide Children's Hospital, Abigail Wexner Research Institute at Nationwide Children's Hospital, Center for Perinatal Research, The Ohio State University Wexner Medical Center, and Riverside Methodist Hospital	Columbus, OH	Non-Author Contributor	
Jonathan L.	Slaughter		MD MPH	Nationwide Children's Hospital, Abigail Wexner Research Institute at Nationwide Children's Hospital, Center for Perinatal Research, The Ohio State University Wexner Medical Center, and Riverside Methodist Hospital	Columbus, OH	Non-Author Contributor	
Sudarshan R.	Jadcherla		MD	Nationwide Children's Hospital, Abigail Wexner Research Institute at Nationwide Children's Hospital, Center for Perinatal Research, The Ohio State University Wexner Medical Center, and Riverside Methodist Hospital	Columbus, OH	Non-Author Contributor	
Patricia	Luzader		RN	Nationwide Children's Hospital, Abigail Wexner Research Institute at Nationwide Children's Hospital, Center for Perinatal Research, The Ohio State University Wexner Medical Center, and Riverside Methodist Hospital	Columbus, OH	Non-Author Contributor	

Supplemental Online Content: Nonauthor Collaborators
*First name, last name, and suffix (if applicable) are required and will appear in PubMed.

***First Name and Middle Initial(s)**	***Last Name**	***Suffix (eg, Jr, III)**	Academic Degrees	Institution	Location (city, state/province, country)	Role or Contribution, eg, chair, principal investigator	Group (if more than 1 Group listed in the byline) and/or Subgroup (eg, Steering Committee)
Julie	Gutentag		RN BSN	Nationwide Children's Hospital, Abigail Wexner Research Institute at Nationwide Children's Hospital, Center for Perinatal Research, The Ohio State University Wexner Medical Center, and Riverside Methodist Hospital	Columbus, OH	Non-Author Contributor	
Erna	Clark		BA	Nationwide Children's Hospital, Abigail Wexner Research Institute at Nationwide Children's Hospital, Center for Perinatal Research, The Ohio State University Wexner Medical Center, and Riverside Methodist Hospital	Columbus, OH	Non-Author Contributor	
Rox Ann	Sullivan		RN BSN	Nationwide Children's Hospital, Abigail Wexner Research Institute at Nationwide Children's Hospital, Center for Perinatal Research, The Ohio State University Wexner Medical Center, and Riverside Methodist Hospital	Columbus, OH	Non-Author Contributor	
Jacqueline	McCool			Nationwide Children's Hospital, Abigail Wexner Research Institute at Nationwide Children's Hospital, Center for Perinatal Research, The Ohio State University Wexner Medical Center, and Riverside Methodist Hospital	Columbus, OH	Non-Author Contributor	

Supplemental Online Content: Nonauthor Collaborators
*First name, last name, and suffix (if applicable) are required and will appear in PubMed.

***First Name and Middle Initial(s)**	***Last Name**	***Suffix (eg, Jr, III)**	Academic Degrees	Institution	Location (city, state/province, country)	Role or Contribution, eg, chair, principal investigator	Group (if more than 1 Group listed in the byline) and/or Subgroup (eg, Steering Committee)
Melanie	Stein		RRT BBA	Nationwide Children's Hospital, Abigail Wexner Research Institute at Nationwide Children's Hospital, Center for Perinatal Research, The Ohio State University Wexner Medical Center, and Riverside Methodist Hospital	Columbus, OH	Non-Author Contributor	
Jennifer L.	Grothause		BA RN BSN	Nationwide Children's Hospital, Abigail Wexner Research Institute at Nationwide Children's Hospital, Center for Perinatal Research, The Ohio State University Wexner Medical Center, and Riverside Methodist Hospital	Columbus, OH	Non-Author Contributor	
Jessica	Purnell		BS CCRC	Nationwide Children's Hospital, Abigail Wexner Research Institute at Nationwide Children's Hospital, Center for Perinatal Research, The Ohio State University Wexner Medical Center, and Riverside Methodist Hospital	Columbus, OH	Non-Author Contributor	
Margaret	Sullivan		BA	Nationwide Children's Hospital, Abigail Wexner Research Institute at Nationwide Children's Hospital, Center for Perinatal Research, The Ohio State University Wexner Medical Center, and Riverside Methodist Hospital	Columbus, OH	Non-Author Contributor	

Supplemental Online Content: Nonauthor Collaborators
*First name, last name, and suffix (if applicable) are required and will appear in PubMed.

***First Name and Middle Initial(s)**	***Last Name**	***Suffix (eg, Jr, III)**	Academic Degrees	Institution	Location (city, state/province, country)	Role or Contribution, eg, chair, principal investigator	Group (if more than 1 Group listed in the byline) and/or Subgroup (eg, Steering Committee)
Julie	Shadd		BA	Nationwide Children's Hospital, Abigail Wexner Research Institute at Nationwide Children's Hospital, Center for Perinatal Research, The Ohio State University Wexner Medical Center, and Riverside Methodist Hospital	Columbus, OH	Non-Author Contributor	
Courtney	Park		RN	Nationwide Children's Hospital, Abigail Wexner Research Institute at Nationwide Children's Hospital, Center for Perinatal Research, The Ohio State University Wexner Medical Center, and Riverside Methodist Hospital	Columbus, OH	Non-Author Contributor	
Hallie	Baugher		BS MSN	Nationwide Children's Hospital, Abigail Wexner Research Institute at Nationwide Children's Hospital, Center for Perinatal Research, The Ohio State University Wexner Medical Center, and Riverside Methodist Hospital	Columbus, OH	Non-Author Contributor	
Demi R.	Beckford		MHS	Nationwide Children's Hospital, Abigail Wexner Research Institute at Nationwide Children's Hospital, Center for Perinatal Research, The Ohio State University Wexner Medical Center, and Riverside Methodist Hospital	Columbus, OH	Non-Author Contributor	

Supplemental Online Content: Nonauthor Collaborators
*First name, last name, and suffix (if applicable) are required and will appear in PubMed.

***First Name and Middle Initial(s)**	***Last Name**	***Suffix (eg, Jr, III)**	Academic Degrees	Institution	Location (city, state/province, country)	Role or Contribution, eg, chair, principal investigator	Group (if more than 1 Group listed in the byline) and/or Subgroup (eg, Steering Committee)
Laura	Marzec		MD	Nationwide Children's Hospital, Abigail Wexner Research Institute at Nationwide Children's Hospital, Center for Perinatal Research, The Ohio State University Wexner Medical Center, and Riverside Methodist Hospital	Columbus, OH	Non-Author Contributor	
Kyrstin	Warnimont		BS	Nationwide Children's Hospital, Abigail Wexner Research Institute at Nationwide Children's Hospital, Center for Perinatal Research, The Ohio State University Wexner Medical Center, and Riverside Methodist Hospital	Columbus, OH	Non-Author Contributor	
Abhik	Das		PhD	RTI International	Research Triangle Park, NC	Non-Author Contributor	
Carla M.	Bann		PhD	RTI International	Research Triangle Park, NC	Non-Author Contributor	
Marie G.	Gantz		PhD	RTI International	Research Triangle Park, NC	Non-Author Contributor	
Jeanette	O'Donnell Auman		BS	RTI International	Research Triangle Park, NC	Non-Author Contributor	
Jenna	Gabrio		BS CCRP	RTI International	Research Triangle Park, NC	Non-Author Contributor	
David	Leblond		BS	RTI International	Research Triangle Park, NC	Non-Author Contributor	
Dhuly	Chowdhury		MS	RTI International	Research Triangle Park, NC	Non-Author Contributor	
Dennis	Wallace		PhD	RTI International	Research Triangle Park, NC	Non-Author Contributor	

Supplemental Online Content: Nonauthor Collaborators
*First name, last name, and suffix (if applicable) are required and will appear in PubMed.

***First Name and Middle Initial(s)**	***Last Name**	***Suffix (eg, Jr, III)**	Academic Degrees	Institution	Location (city, state/province, country)	Role or Contribution, eg, chair, principal investigator	Group (if more than 1 Group listed in the byline) and/or Subgroup (eg, Steering Committee)
Kristin M.	Zaterka-Baxter		RN BSN CCRP	RTI International	Research Triangle Park, NC	Non-Author Contributor	
David K.	Stevenson		MD	Stanford University, El Camino Hospital, and Lucile Packard Children's Hospital	Stanford, Mountain View, and Palo Alto, CA	Non-Author Contributor	
M. Bethany	Ball		BS CCRC	Stanford University, El Camino Hospital, and Lucile Packard Children's Hospital	Stanford, Mountain View, and Palo Alto, CA	Non-Author Contributor	
Melinda S.	Proud		RCP	Stanford University, El Camino Hospital, and Lucile Packard Children's Hospital	Stanford, Mountain View, and Palo Alto, CA	Non-Author Contributor	
Elizabeth N.	Reichert		MA CCRC	Stanford University, El Camino Hospital, and Lucile Packard Children's Hospital	Stanford, Mountain View, and Palo Alto, CA	Non-Author Contributor	
Dharshi	Sivakumar		MD	Stanford University, El Camino Hospital, and Lucile Packard Children's Hospital	Stanford, Mountain View, and Palo Alto, CA	Non-Author Contributor	
Jordan	Williams		BA	Stanford University, El Camino Hospital, and Lucile Packard Children's Hospital	Stanford, Mountain View, and Palo Alto, CA	Non-Author Contributor	
Waldemar A.	Carlo		MD	University of Alabama at Birmingham Health System and Children's Hospital of Alabama	Birmingham, AL	Non-Author Contributor	
Namasivayam	Ambalavanan		MD	University of Alabama at Birmingham Health System and Children's Hospital of Alabama	Birmingham, AL	Non-Author Contributor	
Monica V.	Collins		RN BSN MaEd	University of Alabama at Birmingham Health System and Children's Hospital of Alabama	Birmingham, AL	Non-Author Contributor	

Supplemental Online Content: Nonauthor Collaborators
*First name, last name, and suffix (if applicable) are required and will appear in PubMed.

***First Name and Middle Initial(s)**	***Last Name**	***Suffix (eg, Jr, III)**	Academic Degrees	Institution	Location (city, state/province, country)	Role or Contribution, eg, chair, principal investigator	Group (if more than 1 Group listed in the byline) and/or Subgroup (eg, Steering Committee)
Shirley S.	Cosby		RN BSN	University of Alabama at Birmingham Health System and Children's Hospital of Alabama	Birmingham, AL	Non-Author Contributor	
Cindie	Buie		RN BSN	University of Alabama at Birmingham Health System and Children's Hospital of Alabama	Birmingham, AL	Non-Author Contributor	
Sharon	Owen		RN ADN	University of Alabama at Birmingham Health System and Children's Hospital of Alabama	Birmingham, AL	Non-Author Contributor	
Tara E.	McNair		RN BSN	University of Alabama at Birmingham Health System and Children's Hospital of Alabama	Birmingham, AL	Non-Author Contributor	
Edward F.	Bell		MD	University of Iowa and Sanford Health	Iowa City, IA	Non-Author Contributor	
Tarah T.	Colaizy		MD MPH	University of Iowa and Sanford Health	Iowa City, IA	Non-Author Contributor	
Karen J.	Johnson		RN BSN	University of Iowa and Sanford Health	Iowa City, IA	Non-Author Contributor	
Jacky R.	Walker		RN	University of Iowa and Sanford Health	Iowa City, IA	Non-Author Contributor	
Claire A.	Goeke		DNP ARNP	University of Iowa and Sanford Health	Iowa City, IA	Non-Author Contributor	
Mendi L.	Schmelzel		MSN RN	University of Iowa and Sanford Health	Iowa City, IA	Non-Author Contributor	
Sarah E.	Faruqui		MSN RN	University of Iowa and Sanford Health	Iowa City, IA	Non-Author Contributor	
Brenda J.	Coulter		RN	University of Iowa and Sanford Health	Iowa City, IA	Non-Author Contributor	
Michelle L.	Baack		MD	University of Iowa and Sanford Health	Iowa City, IA	Non-Author Contributor	

Supplemental Online Content: Nonauthor Collaborators
*First name, last name, and suffix (if applicable) are required and will appear in PubMed.

***First Name and Middle Initial(s)**	***Last Name**	***Suffix (eg, Jr, III)**	Academic Degrees	Institution	Location (city, state/province, country)	Role or Contribution, eg, chair, principal investigator	Group (if more than 1 Group listed in the byline) and/or Subgroup (eg, Steering Committee)
Chelsey	Elenkiwich		NNP APRN CNP	University of Iowa and Sanford Health	Iowa City, IA	Non-Author Contributor	
Megan M.	Henning		RN BSN	University of Iowa and Sanford Health	Iowa City, IA	Non-Author Contributor	
Megan	Broadbent		RN BSN	University of Iowa and Sanford Health	Iowa City, IA	Non-Author Contributor	
Sarah	Van Muyden		RN BSN	University of Iowa and Sanford Health	Iowa City, IA	Non-Author Contributor	
Kristi L.	Watterberg		MD	University of New Mexico Health Sciences Center	Albuquerque, NM	Non-Author Contributor	
Janell	Fuller		MD	University of New Mexico Health Sciences Center	Albuquerque, NM	Non-Author Contributor	
Robin K.	Ohls		MD	University of New Mexico Health Sciences Center	Albuquerque, NM	Non-Author Contributor	
Conra	Backstrom Lacy		RN	University of New Mexico Health Sciences Center	Albuquerque, NM	Non-Author Contributor	
Mary	Hanson		RN BSN	University of New Mexico Health Sciences Center	Albuquerque, NM	Non-Author Contributor	
Elizabeth	Kuan		RN BSN	University of New Mexico Health Sciences Center	Albuquerque, NM	Non-Author Contributor	
Sandra	Sundquist Beauman		MSN RNC-NIC	University of New Mexico Health Sciences Center	Albuquerque, NM	Non-Author Contributor	
Sara B.	DeMauro		MD MSCE	University of Pennsylvania, Hospital of the University of Pennsylvania, Pennsylvania Hospital, Children's Hospital of Philadelphia, and Virtua Voorhees Hospital	Philadelphia, PA	Non-Author Contributor	

*First name, last name, and suffix (if applicable) are required and will appear in PubMed.

***First Name and Middle Initial(s)**	***Last Name**	***Suffix (eg, Jr, III)**	Academic Degrees	Institution	Location (city, state/province, country)	Role or Contribution, eg, chair, principal investigator	Group (if more than 1 Group listed in the byline) and/or Subgroup (eg, Steering Committee)
Eric C.	Eichenwald		MD	University of Pennsylvania, Hospital of the University of Pennsylvania, Pennsylvania Hospital, Children's Hospital of Philadelphia, and Virtua Voorhees Hospital	Philadelphia, PA	Non-Author Contributor	
Barbara	Schmidt		MD MSc	University of Pennsylvania, Hospital of the University of Pennsylvania, Pennsylvania Hospital, Children's Hospital of Philadelphia, and Virtua Voorhees Hospital	Philadelphia, PA	Non-Author Contributor	
Haresh	Kirpalani		MB MSc	University of Pennsylvania, Hospital of the University of Pennsylvania, Pennsylvania Hospital, Children's Hospital of Philadelphia, and Virtua Voorhees Hospital	Philadelphia, PA	Non-Author Contributor	
Soraya	Abbasi		MD	University of Pennsylvania, Hospital of the University of Pennsylvania, Pennsylvania Hospital, Children's Hospital of Philadelphia, and Virtua Voorhees Hospital	Philadelphia, PA	Non-Author Contributor	
Aasma S.	Chaudhary		BS RRT	University of Pennsylvania, Hospital of the University of Pennsylvania, Pennsylvania Hospital, Children's Hospital of Philadelphia, and Virtua Voorhees Hospital	Philadelphia, PA	Non-Author Contributor	

*First name, last name, and suffix (if applicable) are required and will appear in PubMed.

***First Name and Middle Initial(s)**	***Last Name**	***Suffix (eg, Jr, III)**	Academic Degrees	Institution	Location (city, state/province, country)	Role or Contribution, eg, chair, principal investigator	Group (if more than 1 Group listed in the byline) and/or Subgroup (eg, Steering Committee)
Christine	Catts		CRNP	University of Pennsylvania, Hospital of the University of Pennsylvania, Pennsylvania Hospital, Children's Hospital of Philadelphia, and Virtua Voorhees Hospital	Philadelphia, PA	Non-Author Contributor	
Megan A.	Dhawan		MSN CRNP	University of Pennsylvania, Hospital of the University of Pennsylvania, Pennsylvania Hospital, Children's Hospital of Philadelphia, and Virtua Voorhees Hospital	Philadelphia, PA	Non-Author Contributor	
Sarvin	Ghavam		MD	University of Pennsylvania, Hospital of the University of Pennsylvania, Pennsylvania Hospital, Children's Hospital of Philadelphia, and Virtua Voorhees Hospital	Philadelphia, PA	Non-Author Contributor	
Toni	Mancini		RN BSN CCRC	University of Pennsylvania, Hospital of the University of Pennsylvania, Pennsylvania Hospital, Children's Hospital of Philadelphia, and Virtua Voorhees Hospital	Philadelphia, PA	Non-Author Contributor	
Jonathan	Snyder		RN BSN	University of Pennsylvania, Hospital of the University of Pennsylvania, Pennsylvania Hospital, Children's Hospital of Philadelphia, and Virtua Voorhees Hospital	Philadelphia, PA	Non-Author Contributor	

Supplemental Online Content: Nonauthor Collaborators
*First name, last name, and suffix (if applicable) are required and will appear in PubMed.

***First Name and Middle Initial(s)**	***Last Name**	***Suffix (eg, Jr, III)**	Academic Degrees	Institution	Location (city, state/province, country)	Role or Contribution, eg, chair, principal investigator	Group (if more than 1 Group listed in the byline) and/or Subgroup (eg, Steering Committee)
Carl T.	D'Angio		MD	University of Rochester Medical Center, Golisano Children's Hospital, and the University at Buffalo John R. Oishei Chidren's Hospital of Buffalo	Rochester and Buffalo, NY	Non-Author Contributor	
Ronnie	Guillet		MD PhD	University of Rochester Medical Center, Golisano Children's Hospital, and the University at Buffalo John R. Oishei Chidren's Hospital of Buffalo	Rochester and Buffalo, NY	Non-Author Contributor	
Anne Marie	Reynolds		MD MPH	University of Rochester Medical Center, Golisano Children's Hospital, and the University at Buffalo John R. Oishei Chidren's Hospital of Buffalo	Rochester and Buffalo, NY	Non-Author Contributor	
Michael G.	Sacilowski		MAT CCRC	University of Rochester Medical Center, Golisano Children's Hospital, and the University at Buffalo John R. Oishei Chidren's Hospital of Buffalo	Rochester and Buffalo, NY	Non-Author Contributor	
Mary	Rowan		RN	University of Rochester Medical Center, Golisano Children's Hospital, and the University at Buffalo John R. Oishei Chidren's Hospital of Buffalo	Rochester and Buffalo, NY	Non-Author Contributor	
Daisy	Rochez		BS MHA	University of Rochester Medical Center, Golisano Children's Hospital, and the University at Buffalo John R. Oishei Chidren's Hospital of Buffalo	Rochester and Buffalo, NY	Non-Author Contributor	

*First name, last name, and suffix (if applicable) are required and will appear in PubMed.

***First Name and Middle Initial(s)**	***Last Name**	***Suffix (eg, Jr, III)**	Academic Degrees	Institution	Location (city, state/province, country)	Role or Contribution, eg, chair, principal investigator	Group (if more than 1 Group listed in the byline) and/or Subgroup (eg, Steering Committee)
Diane I.	Prinzing		AAS	University of Rochester Medical Center, Golisano Children's Hospital, and the University at Buffalo John R. Oishei Chidren's Hospital of Buffalo	Rochester and Buffalo, NY	Non-Author Contributor	
Kyle	Binion		BS	University of Rochester Medical Center, Golisano Children's Hospital, and the University at Buffalo John R. Oishei Chidren's Hospital of Buffalo	Rochester and Buffalo, NY	Non-Author Contributor	
Elizabeth	Boylin		BA	University of Rochester Medical Center, Golisano Children's Hospital, and the University at Buffalo John R. Oishei Chidren's Hospital of Buffalo	Rochester and Buffalo, NY	Non-Author Contributor	
Rosemary L.	Jensen			University of Rochester Medical Center, Golisano Children's Hospital, and the University at Buffalo John R. Oishei Chidren's Hospital of Buffalo	Rochester and Buffalo, NY	Non-Author Contributor	
Rachel	Jones			University of Rochester Medical Center, Golisano Children's Hospital, and the University at Buffalo John R. Oishei Chidren's Hospital of Buffalo	Rochester and Buffalo, NY	Non-Author Contributor	
Constance	Orne			University of Rochester Medical Center, Golisano Children's Hospital, and the University at Buffalo John R. Oishei Chidren's Hospital of Buffalo	Rochester and Buffalo, NY	Non-Author Contributor	

Supplemental Online Content: Nonauthor Collaborators
*First name, last name, and suffix (if applicable) are required and will appear in PubMed.

***First Name and Middle Initial(s)**	***Last Name**	***Suffix (eg, Jr, III)**	Academic Degrees	Institution	Location (city, state/province, country)	Role or Contribution, eg, chair, principal investigator	Group (if more than 1 Group listed in the byline) and/or Subgroup (eg, Steering Committee)
Premini	Sabaratnam		MPH	University of Rochester Medical Center, Golisano Children's Hospital, and the University at Buffalo John R. Oishei Chidren's Hospital of Buffalo	Rochester and Buffalo, NY	Non-Author Contributor	
Ann Marie	Scorsone		MS CCRC	University of Rochester Medical Center, Golisano Children's Hospital, and the University at Buffalo John R. Oishei Chidren's Hospital of Buffalo	Rochester and Buffalo, NY	Non-Author Contributor	
Holly I.M.	Wadkins		MA	University of Rochester Medical Center, Golisano Children's Hospital, and the University at Buffalo John R. Oishei Chidren's Hospital of Buffalo	Rochester and Buffalo, NY	Non-Author Contributor	
Satyan	Lakshminrusimha		MD	University of Rochester Medical Center, Golisano Children's Hospital, and the University at Buffalo John R. Oishei Chidren's Hospital of Buffalo	Rochester and Buffalo, NY	Non-Author Contributor	
Stephanie	Guilford		BS	University of Rochester Medical Center, Golisano Children's Hospital, and the University at Buffalo John R. Oishei Chidren's Hospital of Buffalo	Rochester and Buffalo, NY	Non-Author Contributor	
Emily	Li		BA	University of Rochester Medical Center, Golisano Children's Hospital, and the University at Buffalo John R. Oishei Chidren's Hospital of Buffalo	Rochester and Buffalo, NY	Non-Author Contributor	

Supplemental Online Content: Nonauthor Collaborators
*First name, last name, and suffix (if applicable) are required and will appear in PubMed.

***First Name and Middle Initial(s)**	***Last Name**	***Suffix (eg, Jr, III)**	Academic Degrees	Institution	Location (city, state/province, country)	Role or Contribution, eg, chair, principal investigator	Group (if more than 1 Group listed in the byline) and/or Subgroup (eg, Steering Committee)
Jennifer	Kachelmeyer		BS	University of Rochester Medical Center, Golisano Children's Hospital, and the University at Buffalo John R. Oishei Chidren's Hospital of Buffalo	Rochester and Buffalo, NY	Non-Author Contributor	
Alison	Kent		BMBS FRACP MD	University of Rochester Medical Center, Golisano Children's Hospital, and the University at Buffalo John R. Oishei Chidren's Hospital of Buffalo	Rochester and Buffalo, NY	Non-Author Contributor	
Myra H.	Wyckoff		MD	University of Texas Southwestern Medical Center, Parkland Health & Hospital System, and Children's Medical Center Dallas	Dallas, TX	Non-Author Contributor	
Luc P.	Brion		MD	University of Texas Southwestern Medical Center, Parkland Health & Hospital System, and Children's Medical Center Dallas	Dallas, TX	Non-Author Contributor	
Diana M.	Vasil		MSN BSN RNC-NIC	University of Texas Southwestern Medical Center, Parkland Health & Hospital System, and Children's Medical Center Dallas	Dallas, TX	Non-Author Contributor	
Maria M.	De Leon		RN BSN	University of Texas Southwestern Medical Center, Parkland Health & Hospital System, and Children's Medical Center Dallas	Dallas, TX	Non-Author Contributor	
Joann	Duran		MSN RN BSN	University of Texas Southwestern Medical Center, Parkland Health & Hospital System, and Children's Medical Center Dallas	Dallas, TX	Non-Author Contributor	

Supplemental Online Content: Nonauthor Collaborators
*First name, last name, and suffix (if applicable) are required and will appear in PubMed.

***First Name and Middle Initial(s)**	***Last Name**	***Suffix (eg, Jr, III)**	Academic Degrees	Institution	Location (city, state/province, country)	Role or Contribution, eg, chair, principal investigator	Group (if more than 1 Group listed in the byline) and/or Subgroup (eg, Steering Committee)
Francis	Eubanks		RN BSN	University of Texas Southwestern Medical Center, Parkland Health & Hospital System, and Children's Medical Center Dallas	Dallas, TX	Non-Author Contributor	
Pollieanna	Sepulveda		RN BSN	University of Texas Southwestern Medical Center, Parkland Health & Hospital System, and Children's Medical Center Dallas	Dallas, TX	Non-Author Contributor	
Michelle	Harrod		MSN RN BSN	University of Texas Southwestern Medical Center, Parkland Health & Hospital System, and Children's Medical Center Dallas	Dallas, TX	Non-Author Contributor	
Bradley A.	Yoder		MD	University of Utah Medical Center, Intermountain Medical Center, McKay-Dee Hospital, Utah Valley Hospital, and Primary Children's Medical Center	Murray, Ogden, Provo, and Salt Lake City, UT	Non-Author Contributor	
Mariana	Baserga		MD MSCI	University of Utah Medical Center, Intermountain Medical Center, McKay-Dee Hospital, Utah Valley Hospital, and Primary Children's Medical Center	Murray, Ogden, Provo, and Salt Lake City, UT	Non-Author Contributor	
Stephen. D.	Minton		MD	University of Utah Medical Center, Intermountain Medical Center, McKay-Dee Hospital, Utah Valley Hospital, and Primary Children's Medical Center	Murray, Ogden, Provo, and Salt Lake City, UT	Non-Author Contributor	

Supplemental Online Content: Nonauthor Collaborators
*First name, last name, and suffix (if applicable) are required and will appear in PubMed.

***First Name and Middle Initial(s)**	***Last Name**	***Suffix (eg, Jr, III)**	Academic Degrees	Institution	Location (city, state/province, country)	Role or Contribution, eg, chair, principal investigator	Group (if more than 1 Group listed in the byline) and/or Subgroup (eg, Steering Committee)
Mark J.	Sheffield		MD	University of Utah Medical Center, Intermountain Medical Center, McKay-Dee Hospital, Utah Valley Hospital, and Primary Children's Medical Center	Murray, Ogden, Provo, and Salt Lake City, UT	Non-Author Contributor	
Carrie A.	Rau		RN BSN CCRC	University of Utah Medical Center, Intermountain Medical Center, McKay-Dee Hospital, Utah Valley Hospital, and Primary Children's Medical Center	Murray, Ogden, Provo, and Salt Lake City, UT	Non-Author Contributor	
Jill	Burnett		RNC BSN	University of Utah Medical Center, Intermountain Medical Center, McKay-Dee Hospital, Utah Valley Hospital, and Primary Children's Medical Center	Murray, Ogden, Provo, and Salt Lake City, UT	Non-Author Contributor	
Susan	Christensen		RN	University of Utah Medical Center, Intermountain Medical Center, McKay-Dee Hospital, Utah Valley Hospital, and Primary Children's Medical Center	Murray, Ogden, Provo, and Salt Lake City, UT	Non-Author Contributor	
Brandy	Davis		RN BSN	University of Utah Medical Center, Intermountain Medical Center, McKay-Dee Hospital, Utah Valley Hospital, and Primary Children's Medical Center	Murray, Ogden, Provo, and Salt Lake City, UT	Non-Author Contributor	
Kathleen	Coleman		RN	University of Utah Medical Center, Intermountain Medical Center, McKay-Dee Hospital, Utah Valley Hospital, and Primary Children's Medical Center	Murray, Ogden, Provo, and Salt Lake City, UT	Non-Author Contributor	

Supplemental Online Content: Nonauthor Collaborators
*First name, last name, and suffix (if applicable) are required and will appear in PubMed.

***First Name and Middle Initial(s)**	***Last Name**	***Suffix (eg, Jr, III)**	Academic Degrees	Institution	Location (city, state/province, country)	Role or Contribution, eg, chair, principal investigator	Group (if more than 1 Group listed in the byline) and/or Subgroup (eg, Steering Committee)
Jennifer O.	Elmont		RN BSN	University of Utah Medical Center, Intermountain Medical Center, McKay-Dee Hospital, Utah Valley Hospital, and Primary Children's Medical Center	Murray, Ogden, Provo, and Salt Lake City, UT	Non-Author Contributor	
Barbara L.	Francom		RN BSN	University of Utah Medical Center, Intermountain Medical Center, McKay-Dee Hospital, Utah Valley Hospital, and Primary Children's Medical Center	Murray, Ogden, Provo, and Salt Lake City, UT	Non-Author Contributor	
Jamie	Jordan		RN BSN	University of Utah Medical Center, Intermountain Medical Center, McKay-Dee Hospital, Utah Valley Hospital, and Primary Children's Medical Center	Murray, Ogden, Provo, and Salt Lake City, UT	Non-Author Contributor	
Manndi C.	Loertscher		BS CCRP	University of Utah Medical Center, Intermountain Medical Center, McKay-Dee Hospital, Utah Valley Hospital, and Primary Children's Medical Center	Murray, Ogden, Provo, and Salt Lake City, UT	Non-Author Contributor	
Trisha	Marshant		RNC BSN	University of Utah Medical Center, Intermountain Medical Center, McKay-Dee Hospital, Utah Valley Hospital, and Primary Children's Medical Center	Murray, Ogden, Provo, and Salt Lake City, UT	Non-Author Contributor	
Earl	Maxson		RN CCRN	University of Utah Medical Center, Intermountain Medical Center, McKay-Dee Hospital, Utah Valley Hospital, and Primary Children's Medical Center	Murray, Ogden, Provo, and Salt Lake City, UT	Non-Author Contributor	

Supplemental Online Content: Nonauthor Collaborators
*First name, last name, and suffix (if applicable) are required and will appear in PubMed.

***First Name and Middle Initial(s)**	***Last Name**	***Suffix (eg, Jr, III)**	Academic Degrees	Institution	Location (city, state/province, country)	Role or Contribution, eg, chair, principal investigator	Group (if more than 1 Group listed in the byline) and/or Subgroup (eg, Steering Committee)
Kandace M.	McGrath		BS	University of Utah Medical Center, Intermountain Medical Center, McKay-Dee Hospital, Utah Valley Hospital, and Primary Children's Medical Center	Murray, Ogden, Provo, and Salt Lake City, UT	Non-Author Contributor	
Hena G.	Mickelsen		BA	University of Utah Medical Center, Intermountain Medical Center, McKay-Dee Hospital, Utah Valley Hospital, and Primary Children's Medical Center	Murray, Ogden, Provo, and Salt Lake City, UT	Non-Author Contributor	
D. Melody	Parry		RN	University of Utah Medical Center, Intermountain Medical Center, McKay-Dee Hospital, Utah Valley Hospital, and Primary Children's Medical Center	Murray, Ogden, Provo, and Salt Lake City, UT	Non-Author Contributor	
Susan T.	Schaefer		RN BSN RRT	University of Utah Medical Center, Intermountain Medical Center, McKay-Dee Hospital, Utah Valley Hospital, and Primary Children's Medical Center	Murray, Ogden, Provo, and Salt Lake City, UT	Non-Author Contributor	
Katherine	Tice		RN BSN	University of Utah Medical Center, Intermountain Medical Center, McKay-Dee Hospital, Utah Valley Hospital, and Primary Children's Medical Center	Murray, Ogden, Provo, and Salt Lake City, UT	Non-Author Contributor	
Kimberlee	Weaver-Lewis		RN MS	University of Utah Medical Center, Intermountain Medical Center, McKay-Dee Hospital, Utah Valley Hospital, and Primary Children's Medical Center	Murray, Ogden, Provo, and Salt Lake City, UT	Non-Author Contributor	

Supplemental Online Content: Nonauthor Collaborators
*First name, last name, and suffix (if applicable) are required and will appear in PubMed.

***First Name and Middle Initial(s)**	***Last Name**	***Suffix (eg, Jr, III)**	Academic Degrees	Institution	Location (city, state/province, country)	Role or Contribution, eg, chair, principal investigator	Group (if more than 1 Group listed in the byline) and/or Subgroup (eg, Steering Committee)
Kathryn D.	Woodbury		RN BSN	University of Utah Medical Center, Intermountain Medical Center, McKay-Dee Hospital, Utah Valley Hospital, and Primary Children's Medical Center	Murray, Ogden, Provo, and Salt Lake City, UT	Non-Author Contributor	
Seetha	Shankaran		MD	Wayne State University, Hutzel Women's Hospital, and Children's Hospital of Michigan	Detroit, MI	Non-Author Contributor	
Sanjay	Chawla		MD	Wayne State University, Hutzel Women's Hospital, and Children's Hospital of Michigan	Detroit, MI	Non-Author Contributor	
Kirsten	Childs		RN BSN	Wayne State University, Hutzel Women's Hospital, and Children's Hospital of Michigan	Detroit, MI	Non-Author Contributor	
Girija	Natarajan		MD	Wayne State University, Hutzel Women's Hospital, and Children's Hospital of Michigan	Detroit, MI	Non-Author Contributor	
Bogdan	Panaitescu		MD PhD	Wayne State University, Hutzel Women's Hospital, and Children's Hospital of Michigan	Detroit, MI	Non-Author Contributor	
John	Barks		MD	Wayne State University, Hutzel Women's Hospital, and Children's Hospital of Michigan	Detroit, MI	Non-Author Contributor	
Diane F.	White		RRT CCRP	Wayne State University, Hutzel Women's Hospital, and Children's Hospital of Michigan	Detroit, MI	Non-Author Contributor	
