# Supplementary material for: Respiratory Outcomes After Transcatheter vs Surgical Patent Ductus Arteriosus Closure in Preterm Infants
Source: JAMA Netw Open. 2025 Jun 3;8(6):e2513366. doi: 10.1001/jamanetworkopen.2025.13366 (PMC12134952; doi:10.1001/jamanetworkopen.2025.13366)
Supplement: Supplement 3. — Data Sharing Statement [file jamanetwopen-e2513366-s003.pdf]

Data Sharing Statement

Chock. Respiratory Outcomes After Transcatheter vs Surgical Patent Ductus Arteriosus Closure in Preterm Infants. *JAMA Netw Open*. Published June 03, 2025. doi:10.1001/jamanetworkopen.2025.13366

Data
Data available: Yes
Data types: Other (please specify)
Additional Information: Data reported in this paper may be requested through a data use agreement.
How to access data: Further details are available at https://neonatal.rti.org/index.cfm?fuseaction=DataRequest.Home.
When available: With publication

Supporting Documents
Document types: None

Additional Information
Who can access the data: Researchers whose proposed use of the data has been approved.
Types of analyses: For approved analyses.
Mechanisms of data availability: With a signed data data use agreement and after approval of proposal by the NICHD Neonatal Research Network
